# Sequential Superresolution Imaging of Multiple Targets Using a Single Fluorophore

**DOI:** 10.1371/journal.pone.0123941

**Published:** 2015-04-10

**Authors:** Christopher C. Valley, Sheng Liu, Diane S. Lidke, Keith A. Lidke

**Affiliations:** 1 Department of Pathology and Cancer Research and Treatment Center, University of New Mexico, Albuquerque, New Mexico, United States of America; 2 Department of Physics & Astronomy, University of New Mexico, Albuquerque, New Mexico, United States of America; Julius-Maximilians-University Würzburg, GERMANY

## Abstract

Fluorescence superresolution (SR) microscopy, or fluorescence nanoscopy, provides nanometer scale detail of cellular structures and allows for imaging of biological processes at the molecular level. Specific SR imaging methods, such as localization-based imaging, rely on stochastic transitions between on (fluorescent) and off (dark) states of fluorophores. Imaging multiple cellular structures using multi-color imaging is complicated and limited by the differing properties of various organic dyes including their fluorescent state duty cycle, photons per switching event, number of fluorescent cycles before irreversible photobleaching, and overall sensitivity to buffer conditions. In addition, multiple color imaging requires consideration of multiple optical paths or chromatic aberration that can lead to differential aberrations that are important at the nanometer scale. Here, we report a method for sequential labeling and imaging that allows for SR imaging of multiple targets using a single fluorophore with negligible cross-talk between images. Using brightfield image correlation to register and overlay multiple image acquisitions with ~10 nm overlay precision in the *x-y* imaging plane, we have exploited the optimal properties of AlexaFluor647 for dSTORM to image four distinct cellular proteins. We also visualize the changes in co-localization of the epidermal growth factor (EGF) receptor and clathrin upon EGF addition that are consistent with clathrin-mediated endocytosis. These results are the first to demonstrate sequential SR (s-SR) imaging using direct stochastic reconstruction microscopy (dSTORM), and this method for sequential imaging can be applied to any superresolution technique.

## Introduction

Fluorescence superresolution (SR) imaging has the potential to transform cellular imaging, providing nanometer scale detail about cellular structure and the molecular specificity of fluorescent labeling techniques. The most easily accessible (and arguably the highest resolution) method for fluorescence SR imaging is based on localization of single molecules; (f)PALM, (d)STORM, etc. [1–11]. Since single molecules can be localized with a precision much better than the diffraction limit [12], the found locations of the fluorophores that label the sample can be used to reconstruct an image with resolution much better than the diffraction limit. The achievable resolution is only limited by localization precision and labeling density [13] but in practice is typically ~20 nm and ~50 nm in the lateral and axial directions, respectively.

As with any fluorescence imaging method, it is critical for SR techniques to allow for imaging of multiple targets within a single cell. However, fluorophores commonly used for localization-based SR imaging exhibit highly variable properties in standard imaging buffers, including the number of detected photons, equilibrium on/off duty cycle, the number of switching cycles before irreversible photobleaching, and sensitivity to UV light [14,15]. An additionally complicating factor is the optimization of buffer conditions, which varies between fluorophores (for example, see Table 1 from van de Linde et al. [14] and Table 3 from Dempsey et al. [15]). The difficulty in obtaining optimal fluorophore behavior compatible with localization-based SR imaging is evident in the vast amount of work to both characterize fluorophores [14–21] and to advance buffer conditions [22–26]. Collectively, this inherent variability and continued optimization hinders multi-color SR imaging at present.

An ideal probe for single molecule localization-based SR imaging would have the following properties. (*i*) The probe should start in a fluorescence state so that proper labeling can be verified before imaging. (*ii*) The probe should be easily convertible to a dark state and should be easily maintained at a low fluorescence-state duty cycle during imaging. This low "on state" density in any single image frame is required for the best localization. (*iii*) The probe should undergo many dark-fluorescent-dark switching cycles before photobleaching so that missed localizations don’t result in a complete failure to localize the fluorophore. (*iv*) A large number of photons should be emitted during each switching cycle to give good localization precision. (*v*) The photophysics should not be overly sensitive to buffer conditions or laser power.

Currently, AlexaFluor647 (AF647) in the presence of an oxygen scavenging system along with ~10 mM 2-aminoethanethiol (MEA) is the fluorophore that most nearly approximates the ideal described above. Unfortunately, no other fluorophore (with the exception of the spectrally similar Cy5) has nearly the same performance as AF647 [14,15]. This severely limits reliable, high-quality two- or multi-color SR imaging. Instead of continuing to search for better dyes and imaging conditions, hoping to approach the performance of AF647 in other spectral regions, we propose here a method for sequential SR (s-SR) imaging that uses AF647 to label multiple cellular targets in sequence.

We note the recent development of other sequential methods, including DNA-PAINT [27], Exchange-PAINT [28], and sequential multi-target STORM [29,30], that highlight the need for alternatives to multi-color SR using spectrally distinct dyes. While these methods show promise, they also have significant disadvantages. DNA-PAINT [28] utilizes fluorescent oligos that are costly and target oligos that are difficult to conjugate to antibodies. While the use of DNA pairing of oligonucleotides offers a practically limitless combination of target sequences, stochastic switching—which relies on oligo annealing/melting—is inherently sequence dependent, and the use of fluorescent oligos as well as biotin-streptavidin to conjugate them to antibodies raises concerns of non-specific binding within the cell. Moreover, the initial image is inherently in a dark state, and an image is obtained only upon reconstruction, therefore making verification of labeling as well as initial focusing difficult.

A second method for sequential imaging involves fluorophore quenching using NaBH_4_, a strong reducing agent, in combination with STORM imaging [29], which relies on two independent dyes (an activator and reporter dye) in close proximity. This is advantageous in that NaBH_4_ quenching, with a moderate irreversible quenching efficiency of ~85% for AF647 (as well as for Cy5) [31], is sufficient when two active and proximal dyes are required for stochastic switching, but would likely yield significant cross-talk in single-fluorophore dSTORM. Additionally, antibody conjugation, experimental data collection, and data analysis are more complicated in STORM than with single-fluorophore dSTORM SR imaging. In practice, due to the additional antibody labeling requirements, STORM imaging typically involves labeling with primary and secondary antibodies, therefore any sequential imaging scheme would be limited by the availability of primary antibodies derived from different species, or the use of image segmentation [29]. Furthermore, primary/secondary labeling confounds methods for quantification of SR images [32], and leads to an increase in linkage error (i.e. the displacement between the dye and the target) [33]. A third method performs serial labeling and dSTORM imaging in resin-embedded tissue sections by photobleaching remaining fluorophores using high intensity laser illumination prior to re-labeling [30]. However no estimate is given for fluorophore cross-talk, which likely depends on bleaching intensity and times as well as target labeling density.

We describe here a multi-target imaging capability that uses sequential dSTORM imaging of different cellular components, each labeled with AF647 through directly-conjugated primary antibodies. This method allows a potentially limitless number of components to be imaged, each using the highest quality probe available, and circumvents many of the problems with existing sequential imaging techniques.

## Results

**Fig 1:**
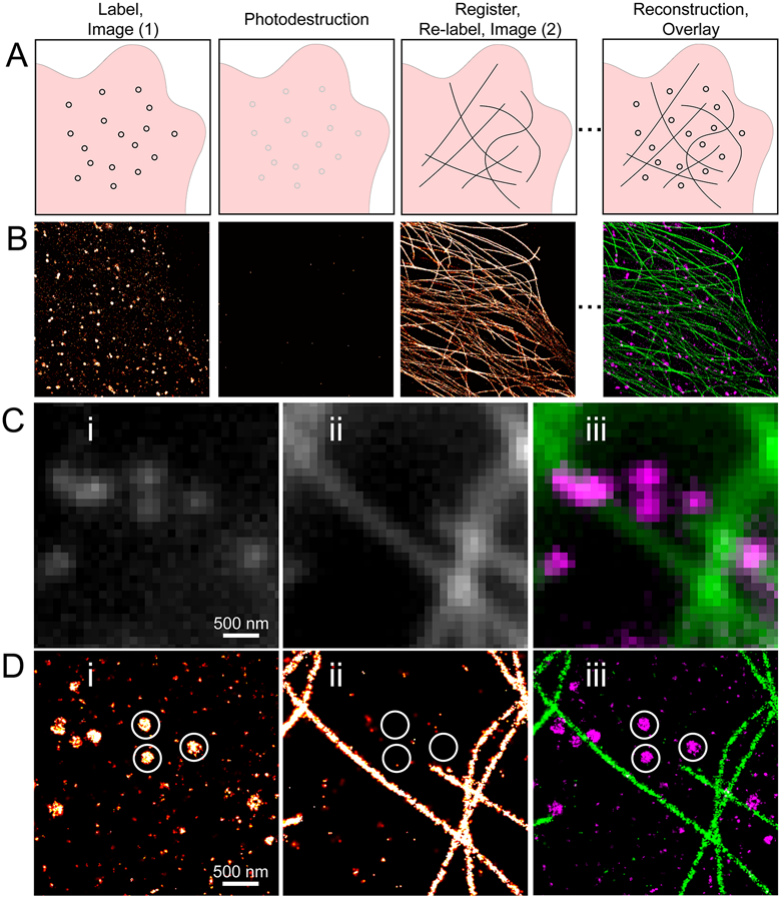
Sequential superresolution imaging. (A) Schematic of sequential imaging, including labeling/imaging, photodestruction, relabeling/imaging, and overlay. Both targets are labeled with the same fluorophore (thus with an identical optical path) and alignment to a reference image is done both prior to and during image acquisition. (B) Sequential imaging of clathrin followed by tubulin, both with AF647, with complete photodestruction between images; including photobleaching and fluorophore quenching (described below). Shown at right is the resulting overlay image of clathrin (magenta) and tubulin (green). (C-D) Zoomed region of sequential imaging of clathrin (left) and tubulin (middle) and the resulting overlay (right—clathrin in magenta, tubulin in green) shown as a diffraction limited image (C) and superresolution reconstruction (D). In the superresolution reconstruction for tubulin, residual localizations from clathrin are notably absent, highlighted by the circles shown. Scale bars 500 nm.

We demonstrate here a method for sequential labeling and SR imaging (sequential superresolution, referred to here as s-SR) that utilizes a combination of photobleaching and irreversible fluorophore quenching—referred to here as “photodestruction” (described below)—to eliminate residual fluorescence signal after SR imaging. Shown schematically in Fig 1A, after imaging and photodestruction, the cell is re-labeled and imaged using a probe (e.g. antibody) conjugated with the same fluorophore. To overlay regions of interest with SR precision, we implement registration via brightfield image correlation [34] that uses cellular contrast to align and stabilize the sample in *x*, *y*, and *z* (see Materials and Methods). Brightfield image correlation was introduced as a means for active stabilization during SR imaging [34]; in addition to stabilization, we use this method to align the sample between imaging of different targets, thus, a multi-target overlay is created without the need for bead registration or overlay algorithms and therefore expedites both image acquisition and analysis. Using s-SR imaging, we were able to image clathrin followed by tubulin, with both structures labeled and imaged using AF647-conjugated primary antibodies (Fig 1B). A zoomed region shows the diffraction limited image (Fig 1C), and the SR reconstruction (Fig 1D). It is worth noting that the small, densely-labeled clathrin structures were non-existent after photodestruction and during imaging of tubulin (Fig 1D, circles). In the remainder of this manuscript, we compare several methods of photodestruction to quantitatively estimate the degree of cross-talk, and we assess the quality of brightfield registration. We further demonstrate sequential labeling and imaging of four different cellular components, and use this method to image the co-localization of EGFR with clathrin-coated pits upon activation by ligand.

### Comparison of fluorescence-reducing methods

Minimizing the fluorescence cross-talk between sequentially imaged structures to an undetectable level is crucial for s-SR imaging of various cellular components. Two easily implemented methods, NaBH_4_ quenching and photobleaching, are investigated in this paper. In standard fluorescence labeling techniques, NaBH_4_ often is used to quench the autofluorescence within the cells that results from fixation using glutaraldehyde. Additionally, Vaughan et al. showed that NaBH_4_ caging of organic dye molecules significantly diminished the fluorescence signal [31]. This caging property of NaBH_4_ can be used to remove the unwanted fluorescence in sequential imaging using STORM [29]. However, NaBH_4_ quenching alone is limited in a s-SR labeling/imaging scheme that utilizes dSTORM for two primary reasons.

**Fig 2:**
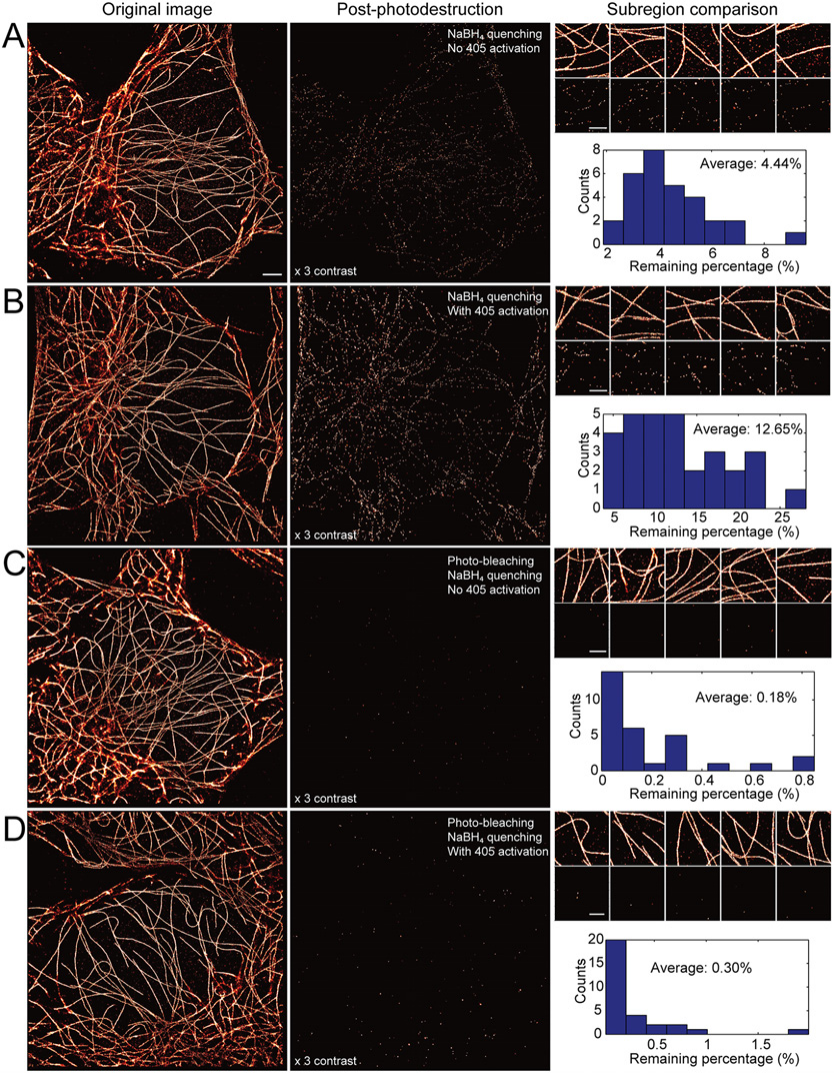
Qualitative and quantitative comparison of different photodestruction methods. (A) Photodestruction with NaBH_4_ quenching alone results in noticeable cross-talk from the original image. Quantification of residual localizations after photodestruction shows an average cross-talk of ~4.5%, from the distribution shown. (B) Photodestruction with NaBH_4_ alone and imaging post-photodestruction done in the presence of low intensity 405 nm excitation shows considerable increase in cross-talk and noticeable residual tubulin structure. Quantitative comparison shows ~13% cross-talk. (C) Photodestruction with both photobleaching and NaBH_4_ quenching shows little detectable cross-talk. Subregion quantification shows cross-talk of ~0.2%, representing a ~20-fold improvement over NaBH_4_ alone. (D) Photodestruction via both photobleaching and NaBH_4_ quenching, and imaging post-photodestruction with 405 nm excitation shows considerable improvement and no residual tubulin structure. Quantitative comparison of residual localizations shows ~0.3% cross-talk, a ~40-fold improvement over NaBH_4_ quenching alone. Therefore photobleaching and NaBH_4_ quenching provides an ideal method for photodestruction in s-SR. Scale bars in original image (left), 2 μm; scale bars in small subregions (right), 1 μm. Note, the image contrast in all post-photodestruction images (middle column) was increased by 3× in order to make visible the any remaining signal. Cross-talk was estimated by calculating residual localization in small, ~2 x 2 μm, subregions after photo-destruction (see Subregion comparison, right column). Data for each histogram represents ~30 subregions taken from at least five independent cells.

First, quenching of fluorophores using NaBH_4_ results in irreversible caging of ~85% of AF647 molecules [31], however, the remaining 15% of fluorophores undergo spontaneous uncaging at a low but measurable rate, resulting in unwanted cross-talk between images collected in sequence. This method of NaBH_4_ is effective for sequential imaging using STORM [29] owing to the nature of requiring two independent and active fluorophores for stochastic blinking, however in dSTORM any spontaneous uncaging would directly result in unwanted cross-talk. To estimate the level of cross-talk due to spontaneous uncaging of fluorophores, we performed dSTORM imaging of α-tubulin with AF647 using a directly conjugated primary antibody (α-tubulin-AF647) in HeLa cells both before (Fig 2A, left) and after NaBH_4_ quenching (Fig 2A, middle). After reconstruction, 30 subregions were selected from the six different cells, and these subregions were used to estimate the percentage of emitters remaining (Fig 2A, right). After NaBH_4_ quenching alone, the percentage of emitters remaining is 4.44% on average, and the distribution of the level of cross-talk is shown in Fig 2A.

Second, the problem of NaBH_4_ quenching alone is confounded by the use of ultraviolet excitation, which causes rapid uncaging of fluorophore molecules not irreversibly caged during NaBH_4_ quenching [31]. This poses a significant problem in that ultraviolet (e.g. 405 nm) excitation is often used to accelerate the rate of stochastic cycling between fluorescent and dark states during dSTORM image acquisition. Therefore, s-SR imaging using dSTORM with additional 405 nm illumination would cause substantial cross-talk from previously imaged targets if fluorophores are only quenched using NaBH_4_. To estimate this cross-talk percentage, we again performed dSTORM imaging of α-tubulin-AF647 in HeLa cells as before, followed by NaBH_4_ quenching and re-imaging in the presence of low intensity illumination (5 W/cm^2^) with a 405 nm laser (Fig 2B). The remaining percentage of fluorophores increases three fold in the presence of 405 illumination, representing cross-talk of ~12.6% between images. This result is consistent with the UV uncaging of NaBH_4_-quenched fluorophores as previously described [31], making this method of photodestruction unsuitable for s-SR imaging.

Another effective method for reducing the fluorescence is through irreversible photobleaching. However, due to the high labeling density of SR samples, photobleaching alone resulted in substantial cross-talk (~7%, see S1 Fig) and is therefore not ideal for s-SR imaging. Although photobleaching time may be increased, we used a combination of photobleaching and NaBH_4_ quenching to minimize overall cross-talk and to shorten the overall time required for elimination of fluorophores. Importantly, prior to photobleaching, the sample was washed several times to eliminate the imaging buffer (which would protect the fluorophore from photobleaching) and was placed into PBS. After 5 minutes of photobleaching for each cell with high intensity 405 nm (~0.24 kW/cm2) and 637 nm (~1.7 kW/cm2) illumination, the sample was quenched for 10 minutes using NaBH_4_. We found this combination of photobleaching and quenching rendered cross-talk negligible, with only ~0.2% cross-talk (Fig 2C). Furthermore, this procedure also significantly lessens the effect caused by UV illumination; when 405 nm illumination was used after photobleaching and quenching, the degree of cross-talk increased only to ~0.3% (Fig 2D). This combination of photobleaching and NaBH_4_ quenching results in near complete elimination of fluorophores and is suitable for s-SR imaging, even in the presence of 405 nm illumination.

We additionally tested s-SR imaging of antibodies conjugated to photocleavable biotin with secondary labeling via AF647-conjugated NeutrAvidin. In this labeling scheme, photolysis occurs with 405 nm excitation, causing direct uncoupling of the fluorophore from the antibody. While photocleavable biotin-labeled anti-α-tubulin gave sufficient SR imaging results with NeutrAvidin-647, we found that photolysis alone yielded substantial cross-talk and residual tubulin structure (S2 Fig). We also found no improvement by including photolysis in addition to photobleaching and quenching as described (S3 Fig). An additional complication was that the use of photocleavable biotin-conjugated antibodies requires a secondary labeling step with AF647-conjugated streptavidin or NeutrAvidin, which adds time to the labeling process and is prone to non-specific binding within cells.

### Colocalization of α-tubulin and β-tubulin

Another critical factor in s-SR imaging is the ability to accurately realign the cell with SR precision. Since the sequential imaging scheme presented here involves buffer exchange and even removal of the sample from the microscope stage, we implemented a robust and rapid method for alignment, re-alignment, and registration of the sample in *x*, *y*, and *z*, using brightfield imaging and cross-correlation analysis similar, but not identical to that previously described [34]. Briefly, a brightfield reference image is collected prior to SR acquisition. This reference image is then used to register the cell in all three dimensions both during image acquisition as a means for active stabilization as well as between different labels as a means for image registration. Image stabilization and registration involves iterative collection of a brightfield z-stack, followed by cross-correlation analysis of each single image within the z-stack for alignment in *z*, and finally a shift in the *x-y* imaging plane (S4 Fig).

**Fig 3:**
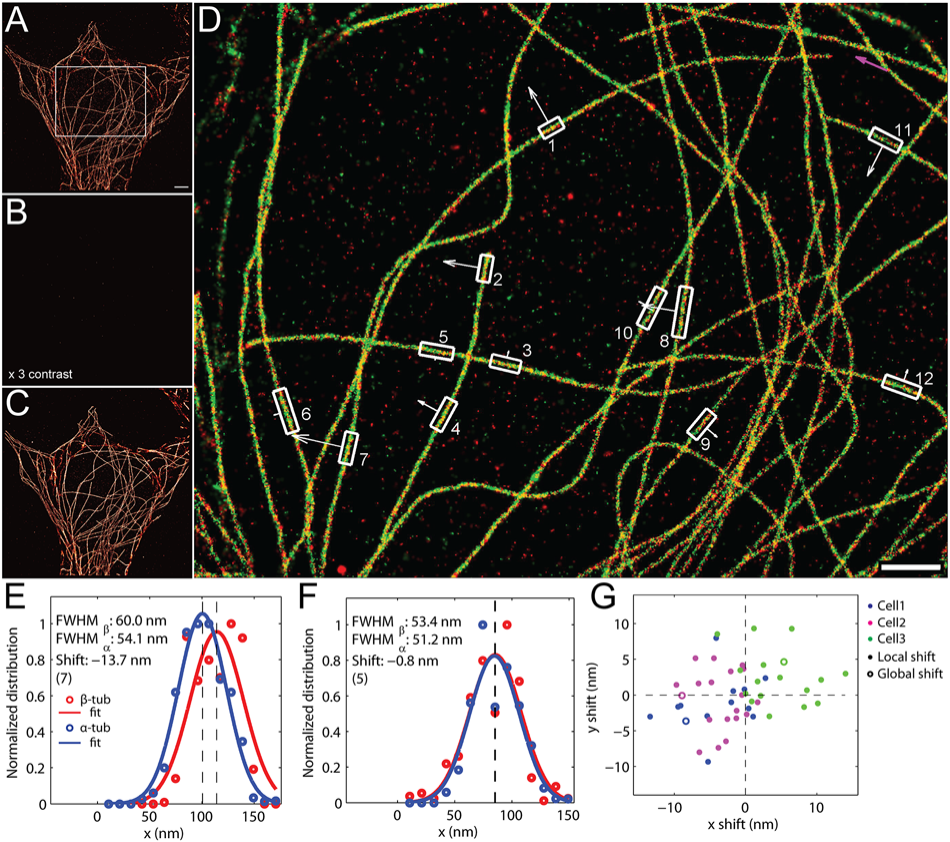
Quantitative analysis of brightfield channel registration. The HeLa cell was labeled and imaged for β-tubulin, scale bar 2 μm (A), followed by photodestruction with bleaching and NaBH_4_ (B). The image contrast in the post-photodestruction image (B) is increased by 3× to highlight the absence of measurable cross-talk. A brighfield, phase contrast reference image of the ROI was collected prior to imaging. The ROI was realigned to this reference image before and during each acquisition. After photodestruction, the cell was re-labeled and imaged for α-tubulin (C), with alignment to the original reference image. (D) The resulting overlay showing β-tubulin (red) and α-tubulin (green) with good qualitative overlay. Scale bar 1 μm. (E-G) Analysis of the entire ROI in (D) as well as small microtubule-containing subregions (D, white boxes) were carried out to quantify global and local shifts in the overlay. For calculation of local shifts, the distribution of localizations along the width of the microtubule (E-F, open circles) was fit to a Gaussian (E-F, solid line) and the shift was taken to be the difference between the estimated mean values of the Gaussian fit for β-tubulin (E-F, red) and α-tubulin (E-F, blue), shown as vertical dashed lines. Shown are the local shifts taken from Box 7 (E) and Box 5 (F) from the ROI shown in (D). (G) The resulting global shift (open circle) and local shifts (closed circles) in *x* and *y* for three independent cells.

In order to test the reliability and to estimate the precision of the brightfield alignment, we performed s-SR imaging of β- and α-tubulin, each directly conjugated with AF647, in the same cell for multiple cells. Fig 3A shows the SR reconstruction of anti-β-tubulin-AF647 labeling in one of the imaged cells. Photodestruction in each cell was performed using a combination of photobleaching and NaBH_4_ quenching as described above, and the reconstruction after photodestruction is nearly blank, indicating negligible cross-talk (Fig 3B). The cell was subsequently washed and labeled with anti-α-tubulin-AF647 for 2 hours, followed by washing. Prior to and periodically during re-imaging of α-tubulin, the region of interest was aligned using brightfield registration of the cell within the field of view, using the brightfield reference image collected prior to imaging β-tubulin. The reconstruction of α-tubulin in the corresponding cell is shown in Fig 3C. The resulting overlay image of β-tubulin (red) and α-tubulin (green) is shown in Fig 3D. No noticeable shift exists between the two structures in the overlay image, indicating qualitatively acceptable degree of alignment and registration.

To quantitatively estimate the alignment precision, we compared the reconstructed images of β- and α-tubulin over the entire region of interest as well as many subregions throughout each image. We first estimated any global shift between the two tubulin labels over the entire region of interest, which may be caused by error in registration/alignment causing a unidirectional shift between the two tubulin components. The magnitude of the global shift for the complete image shown in Fig 3D is ~9.1 nm (-8.3 nm shift in *x*, -3.6 nm shift in *y*). The purple arrow (Fig 3D, top right) shows the direction and relative magnitude of this global shift (note that the origin of the figure is in the top-left corner). This global shift value is also shown as blue, open circle in Fig 3G (corresponding to Cell 1).

In addition to global shifts caused by overall error in the overlay, there may exist local shifts due to subtle changes in cell morphology that occurred between imaging of β- and α-tubulin. To determine the extent of any local shift, subregions were drawn around approximately linear fragments of tubulin (Fig 3D, white boxes), and the distribution of localizations along the microtubule width dimension (summed along the tubulin length dimension) for β- and α-tubulin (Fig 3E and 3F, red and blue circles for β- and α-tubulin, respectively) were fit with a Gaussian function (Fig 3E and 3F, solid red and blue lines for β- and α-tubulin, respectively). The shift between the two microtubules is taken to be the shift between the estimated mean values of the Gaussian fit (Fig 3E and 3F, vertical dashed lines). Shown in Fig 3E and 3F are the distributions of localizations, Gaussian fits, and resulting shifts for box 7 (-11.5 nm) and box 9 (-0.2 nm), respectively, from Fig 3D. The direction of these local shifts (which can be estimated only for the dimension perpendicular to the microtubule) and the relative magnitude of these local shifts are shown as white arrows adjacent to each subregion analyzed (Fig 3D, white boxes and white arrows). The scatter plot in Fig 3G shows the estimated global shifts (open circles) and the local shifts (closed circles) from three independently imaged cells (for additional cells and complete quantification, see S5–S9 Figs). For example, data points for cell 1, shown in blue in the scatter plot, are derived from α- and β-tubulin shown in Fig 3D. The data for cell 2 (Fig 3G, purple points) and cell 3 (Fig 3G, green points) are identically derived from independent cells, with overlay images and subregions for these cells shown in S5–S9 Figs.

The local shifts are consistent with the global shift (i.e. the closed circles and open circle for each cell in Fig 3G are in the same direction) with a few exceptions, largely owing to microtubule orientation relative to the direction of the global shift. For example, the shift in box 4 (Fig 3D) should be comparable to the global shift (Fig 3D, purple arrow) since the microtubule is nearly perpendicular to the global shift axis. Conversely, the shift in boxes 3 and 5 (Fig 3D) should be near zero, because the microtubule is oriented parallel to (and therefore localization are summed along) the global shift dimension. Exceptions may be caused by slight structural changes that occur during sequential imaging, local differences in the localization accuracy of the two structures, or undersampling of the microtubule.

The distribution of local shifts between α- and β-tubulin in microtubule fragments is within ~10 nm (Fig 3G), which indicates an accurate registration of the same cell between the two labeling cycle and an adequate fixation of the cell sample, both of which are critical for s-SR imaging. We note no patterns of local shifts that indicate any sample rotation. Collectively, our s-SR imaging method is capable of realigning the cell with an estimated overlay precision of ~10 nm.

### Sequential superresolution imaging of four cellular components

**Fig 4:**
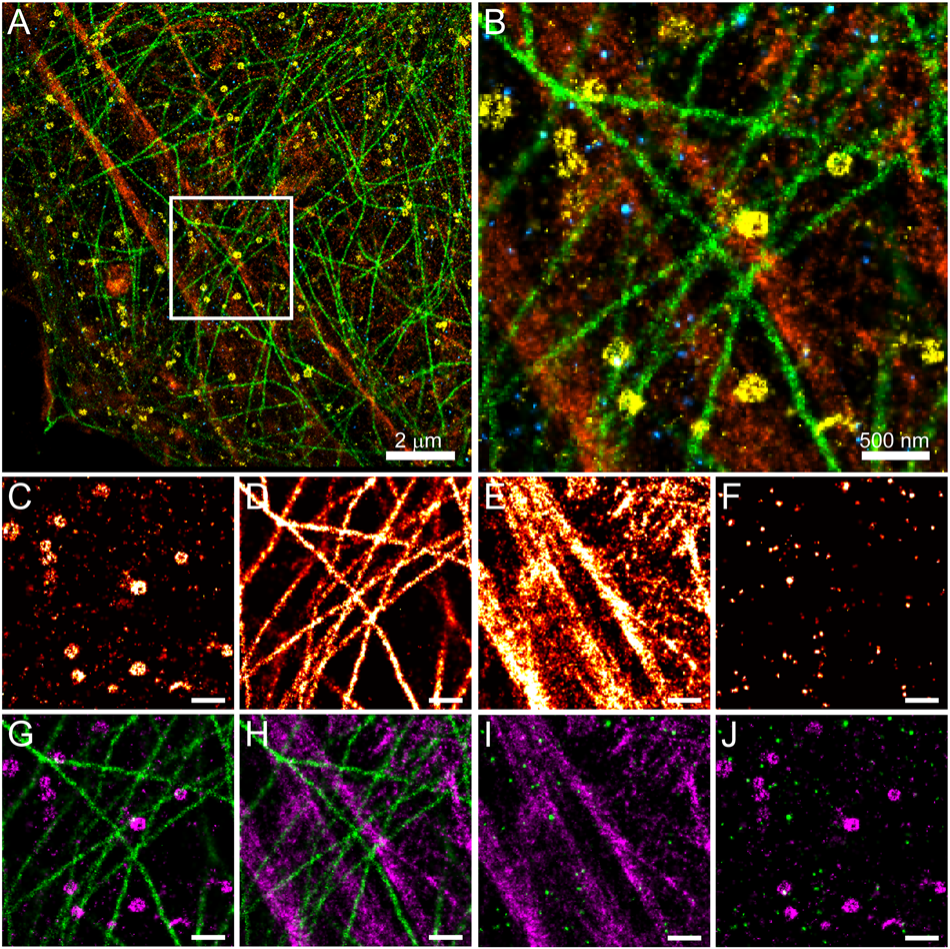
Four color s-SR imaging using a single fluorophore. (A-B) Four color reconstruction overlay showing clathrin (yellow), α-tubulin (green), actin (orange), and EGFR (blue), imaged sequentially in the order listed, with each target imaged with AF647-conjugated primary antibody with the exception of actin, which was imaged with phalloidin-AF647. (B) Zoomed region highlighted in (A). (C-F) The original reconstruction for each individual component—clathrin (C), α-tubulin (D), actin (E), and EGFR (F)—of the region shown in (B) and highlighted in (A), scale bars 500 nm. Note the lack of any measurable cross-talk between images. (G-J) Two-label reconstructions for clathrin and tubulin (G), tubulin and actin (H), actin and EGFR (I), and clathrin and EGFR (J).

In Figs 1 and 3, we demonstrated s-SR imaging of two targets. A major advantage of s-SR imaging using dSTORM is the ability to label various cellular components without the need for consideration of secondary antibodies species. We next sought to sequentially image multiple independent targets, each with a directly conjugated primary antibody, with the exception of actin, which was labeled with phalloidin, and all imaged using AF647. Shown in Fig 4 is the s-SR image reconstruction of four targets with each having a distinct structure in HeLa cells; clathrin, α-tubulin, actin, and the epidermal growth factor receptor (EGFR). HeLa cells were fixed and permeabilized as described (see Materials and Methods). The four color overlay shows distinct labeling of clathrin (Fig 4A and 4B, yellow), α-tubulin (Fig 4A and 4B, green), actin (Fig 4A and 4B, orange), and EGFR (Fig 4A and 4B, blue); and the zoomed region (Fig 4B) shows the organization of each target within a subregion of the cell. Cells were first labeled/imaged with anti-clathrin-AF647 (Fig 4C), followed by anti-α-tubulin-AF647 (Fig 4D), phalloidin-AF647 (Fig 4E), and finally α-EGFR-AF647 (Fig 4F), with alignment to the reference image done before and during image acquisition. The image alignment at the beginning of each target, relative to the initial reference image, is shown in S10 Fig. The near complete absence of cross-talk, based on the lack of detectable signal following photodestruction even in the most densely labeled structures, indicates that the previous target was completely photodestroyed. For example, EGFR—lacking any macromolecular structure—was imaged last, and immediately following actin, as a test of our ability to eliminate any residual signal from actin (phalloidin) labeling. Importantly, we detect no residual signal even in regions which contained large and densely labeled actin stress fibers (compare Fig 4E and 4F). For simplicity, shown in Fig 4G–4J are two-color overlays for clathrin and tubulin (Fig 4G), tubulin and actin (Fig 4H), actin and EGFR (Fig 4I), and clathrin and EGFR (Fig 4J).

### Clathrin mediated endocytosis of EGFR imaged by s-SR

**Fig 5:**
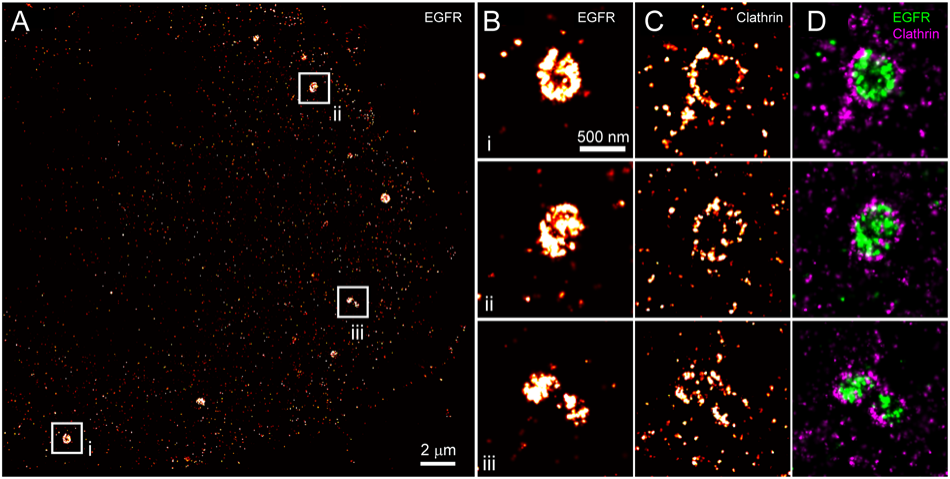
Co-localization of EGFR to clathrin-coated vesicles upon activation. HeLa cells were activated with EGF at 1.5 ng/ml for 10 minutes followed by fixation and labeling with anti-EGFR conjugated to AF647. (A) Activation of EGFR results in the formation of puncta in the plasma membrane. (B) Zoomed regions i-iii illustrate the size of EGFR aggregates. (C) After photodestruction, cells were re-labeled with anti-clathrin-AF647 and imaged. The clathrin reconstruction shows ring-like structures in the proximity of EGFR aggregates. (D) The resulting reconstruction overlay (EGFR in green, clathrin in magenta) clearly shows the formation of clathrin-coated vesicles, with clathrin encircling EGFR within these endosomes. We note that neither EGFR aggregates nor any correlation between EGFR and clathrin was observed in the absence of stimulation by EGF (see Fig 4J and S11 Fig).

We next sought to use s-SR imaging to observe co-localization of EGFR with clathrin-coated pits. Upon activation by EGF ligand, EGFR undergoes rapid internalization and degradation [35–37]. Furthermore, at low concentrations of EGF, clathrin-mediated endocytosis is a primary mechanism for internalization of EGFR, whereby receptors are removed from the plasma membrane via clathrin-coated pits [38,39]. To directly observe EGFR within clathrin-coated pits, we treated HeLa cells with 1.5 ng/ml (~0.25 nM) EGF for 10 minutes at 37°C to maximize clathrin-mediated endocytosis of EGFR relative to other internalization pathways [39]. Cells were immediately fixed and labeled with an anti-EGFR antibody directly conjugated with AF647 and imaged using SR microscopy. Activation of EGFR resulted in the formation of large receptor aggregates, ~200–400 nm in diameter (Fig 5A and 5B). In resting cells the distribution of EGFR was uniform throughout the membrane and showed no obvious correlation with clathrin (Fig 4J and S11 Fig). After imaging of EGFR, cells were subjected to photodestruction and subsequently re-labeled with anti-clathrin directly conjugated with AF647 (Fig 5C). The cell was realigned using brightfield registration and re-imaged again to observe the pattern of clathrin staining around the EGFR aggregates. The reconstructed images of clathrin show a distinct ring-like structure (Fig 5C), and overlay images confirm their localization around EGFR in a pattern consistent with clathrin-mediated endocytosis of EGFR (Fig 5D). We also note the complete lack of cross-talk from the densely-labeled EGFR within the endosome, and also the location of EGFR relative to clathrin is consistent with the N-terminal epitope of the EGFR antibody used in this experiment.

## Discussion

We describe here a novel method for labeling and SR imaging of multiple targets with a single fluorophore—here AF647—which utilizes a combination of photobleaching and quenching, and reduces cross-talk to less than 0.5% between images (Fig 2). The use of low intensity 405 nm illumination, an important component in dSTORM used to facilitate fluorophore recovery to a fluorescent state and accelerate the rate of dark-fluorescent-dark switching cycles, does not substantially increase the level of cross-talk and can therefore be used with any or all targets. Additionally, using brightfield image correlation for registration/alignment and stabilization, we were able to achieve an overlay accuracy of ~10 nm in the *x-y* imaging plane (Fig 3) without the need for fluorescent beads or other physical reference points, even after the sample was removed from the microscope stage entirely. This allows the user to remove the sample from the microscope and therefore “parallelize” image acquisition.

Sequential imaging using a single fluorophore allows one to take advantage of the superior photophysical capabilities of the best available fluorophore for dSTORM imaging, currently AF647, and any found improvements in buffer or imaging conditions, such as those recently described [22,23], benefit all structures. Moreover, advanced image acquisition and analysis approaches, including multi-emitter localization [40–43] and 3D imaging [44–50], that require prior knowledge of the photophysics and/or calibration of the fluorophore point spread function, can be optimized for a single color with a well-known set of parameters. The near lack of cross-talk between targets also makes s-SR ideal for quantitative analysis methods such as pair-correlation analysis [32,51,52] as well as density-based analysis methods [53,54]. Lastly, that the optical path is identical for each target negates any chromatic aberrations between channels.

An additional advantage is that the sequential imaging technique presented here requires minimal equipment and reagents. The majority of SR microscopes are equipped with UV (405 nm) and far-red (~ 635–647 nm) lasers, therefore, without the need for additional high power lasers this setup is a cost-effective method to achieve multi-color SR imaging. Furthermore, conjugation to antibodies need only be optimized for a single fluorophore, rather than a library of oligo-antibody complexes [28], and the use of directly labeled primary antibodies greatly simplifies sample preparation and circumvents limitations due to species of derived antibodies and resulting requirement for image segmentation [29].

The primary disadvantage in our current implementation of s-SR is the total time required for imaging and re-labeling. For example, the entire process for labeling and imaging of two targets (Fig 5) and four targets (Fig 4, for example) required approximately 5 and 10 hours, respectively, and in each case four cells were imaged. However, the total imaging time is highly dependent on optimal antibody labeling durations, the required acquisition time, and the total number of cells imaged. Since each individual cell requires photobleaching in this sequential imaging scheme, the user is limited to cells that were imaged and photobleached during previous imaging cycles (as all cells are quenched, but only selected cells undergo photobleaching). Therefore the process may be accelerated by increasing the number of cells imaged initially and, during subsequent rounds of data acquisition, selectively imaging a subset of those cells that have adequate labeling density and suitable focal position for the current target. Furthermore, the entire process may be automated using a combination of motor-controlled and piezo stages as well as the incorporation of microfluidics devices for buffer exchange, relabeling, and washing [55].

Using s-SR imaging, it is critical that buffers be readily exchanged after image acquisition, by manual pipetting, through automated buffer exchange, or by using a microfluidics system. Therefore this method is incompatible with techniques that require viscous or solidifying mounting media. Additionally, careful pipetting during buffer exchange is recommended to preserve cell morphology and thus to minimize error when using the brightfield registration.

We demonstrated here the use of brightfield registration to achieve an overlay precision in the *x-y* imaging plane of ~10 nm, however this is dependent on cell morphology and likely also fixation protocol. Optimization is required to ensure proper fixation such that cell morphology is not changing throughout the course of labeling, photodestruction, washing, re-labeling, etc. This should be verified, for example by sequential imaging of α- and β-tubulin and application of the brightfield (or other) registration to estimate the overlay error for a given fixation protocol (see Fig 3 and Materials and Methods). The brightfield alignment as described is necessarily performed in 3D and therefore the method can be directly be used for 3D SR imaging using sequential labeling.

In summary, the approach presented here demonstrates a sequential imaging approach that takes advantage of the widely-used dSTORM imaging method. Sequential labeling and imaging of multiple targets with a single fluorophore is a simple and practical alternative to multi-color SR imaging using spectrally distinct labels and other recently developed methods for s-SR imaging [28,29]. Collectively, this allows for imaging of multiple labels at the molecular scale and could additionally be implemented with a variety of SR techniques where multi-color imaging is difficult, including stimulated emission depletion [56], image scanning microscopy [57], and structured illumination microscopy [58].

## Materials and Methods

### Cell lines and reagents

HeLa cells were cultured in Dulbecco’s Modified Eagle Medium (Life Technologies # 10313–021) supplemented with 10% fetal bovine serum (HyClone), penicillin-streptomycin, and 2 mM L-glutamine, and cells were maintained at sub-confluence.

Anti-EGFR-AF647 antibody (anti-EGFR, R-1, sc-101) was purchased from Santa Cruz Biotechnology. Anti-alpha-tubulin (T6074) and anti-beta-tubulin (T8328) antibodies were purchased from Sigma. Anti-clathrin was purchased from Abcam (Clathrin heavy chain, ab21679). All primary antibodies were either purchased pre-conjugated to AF647 or were conjugated directly (see below) using AlexaFluor647 carboxylic acid, succinimidyl ester (A-20006), purchased from Life Technologies, with the exception of anti-clathrin, which was directly conjugated using the APEX AlexaFluor-647 antibody labeling kit (Life Technologies, A10475) due to the presence of carrier proteins. AlexaFluor-647 phalloidin was purchased from Life Technologies (A22287). Sodium borohydride was purchased from EMD (SX0380-3). Photocleavable biotin NHS (PC-biotin-NHS) was purchased from Ambergen, and purified NeutrAvidin protein was purchased from ThermoScientific (31000).

### Antibody conjugation

Conjugations of primary antibodies and NeutrAvidin to AlexaFluor647 was carried in 100–120 μl volume containing 100 mM sodium bicarbonate with 1mg/ml final concentration of antibody and a ~10× molar excess of reactive AF647 to achieve a final ratio of 2–5 dye molecules per antibody. The reaction was carried out for 1 hour at room temperature with constant mixing, protected from light. Conjugations of primary antibodies to PC-biotin were carried in 100–120μl volume with 1 mg/ml final concentration of antibody and a 25 × molar excess of PC-biotin-NHS, and 100mM sodium bicarbonate. The reaction was carried out for approximately one hour at room temperature with constant mixing and protected from light.

Labeled antibodies and NeutrAvidin were purified using gravity columns with Bio-Gel P-30 gel (BioRad #150–4154) to separate antibody from free dye/label. For PC-biotin-conjugated antibody, which is not visible within the column, multiple fractions were collected, and protein concentration was measured using 280 nm-absorbance to determine the fractions that contain antibody. Protein concentration was confirmed by BCA assay (Pierce). Conjugation of AF647 to antibodies containing BSA and/or other carrier proteins was done using the APEX AlexaFluor-647 antibody labeling kit according to the manufacturer instructions.

### Fixation and labeling

For imaging, HeLa cells were seeded onto 25 mm round coverslips (Electron Microscopy Sciences #72196–25) within 6-well plate at 225,000 cells per well, or onto 8-well chamber slides (Lab-Tec, Thermo Scientific #155411) at 20,000 cells per well, and allowed to adhere to the glass overnight.

Cells were washed in pre-warmed PBS and fixed/permeabilized in 0.6% paraformaldehyde + 0.1% glutaraldehude + 0.25% (w/v) Triton X-100 for 60 seconds immediately followed by fixation in 4% paraformaldehyde + 0.2% glutaraldehyde for ~2 hours. Cells were washed extensively with PBS, washed once with 0.1% NaBH_4_ for ~7 minutes to reduce background fluorescence, and once with 10 mM Tris-HCl (pH 7.2) for ~7 minutes to quench reactive cross-linkers. Samples were blocked in PBS + 5% BSA for 15–30 minutes and stored at 4°C in PBS + 2% BSA + 0.05% sodium azide.

Cells were labeled with the indicated antibody in PBS + 2% BSA + 0.05% Triton X-100 followed by extensive washing. For samples labeled with photocleavable biotin conjugated primary antibodies, secondary labeling was carried out using NeutrAvidin-AF647 at 10 μg/ml for 10–15 minutes and washed thoroughly with PBS/BSA. Endogenous biotin-blocking kit (Molecular Probes E-21390) was used before sample blocking to minimize non-specific intracellular binding of NeutrAvidin-AF647.

### Sequential imaging of four cellular components

HeLa cells were plated in 8-well chamber slides and allowed to adhere to the glass overnight. After fixation (as previously described), cells were labeled with anti-clathrin-AF647 at 2.5 μg/ml in PBS + 2% BSA + 0.05% Triton X-100 (PBS/BSA/TX) overnight and washed extensively in PBS/BSA/TX. For each imaged cell, a brightfield reference image was collected. Following SR image acquisition, cells were photobleached and quenched (see below), followed by re-labeling with anti-α-tubulin-AF647 at 2.5 μg/ml for 45 minutes in PBS/BSA/TX followed by extensive washing. Each cell was re-imaged, using the reference image for alignment via brightfield registration (see below). Cells were again photobleached and quenched (see below), followed by re-labeling with Phalloidin-AF647 at ~0.5 μM in PBS for 20 minutes followed by washing with PBS. Each cell was again re-imaged, using the original reference image for alignment. Cells were then photobleached and quenched, followed by labeling with anti-EGFR-AF647 at 5 μg/ml in PBS/BSA/TX for 1 h followed by extensive washing. Each cell was re-imaged, using the reference image for alignment. Shown in S10 Fig are the results of the brightfield alignment at each step, with maximum of cross-correlations > 0.94. Due to the identical optical path for each target, and the high brightfield cross-correlation, no further transformations were needed to generate the overlay image.

### Sample preparation for imaging of clathrin-mediated endocytosis of EGFR

HeLa cells were seeded onto 8-well chamber slides at 20,000 cells per well, and allowed to adhere to the glass overnight. Following serum starvation at 37°C for ~1 h, cells were treated with EGF (LifeTechnologies #PHG0311) at 1.5 ng/ml (~0.25 nM) for 10 min at 37°C [38,39]. Cells were washed once with PBS and immediately fixed in 4% paraformaldehyde + 0.2% glutaraldehyde for ~2 hours. Cells were washed extensively with PBS, washed once with 0.1% NaBH_4_ for ~7 minutes to reduce background fluorescence, and once with 10 mM Tris-HCl (pH 7.2) for ~7 minutes to quench cross-linkers. Samples were blocked and permeabilized in PBS + 5% BSA + 0.1% Triton X-100 for 20 minutes. Cells were first labeled with anti-EGFR-AF647 at 5 μg/ml overnight and imaged, followed by labeling with anti-Clathrin-AF647 at 2.5 μg/ml for 2 hours and re-imaging. All antibody incubations and washes were done in PBS + 2% BSA + 0.05% Triton X-100.

### Optical setup

The imaging system is custom-built from an inverted microscope (IX71, Olympus America Inc.). A *xyz* piezo stage (Mad City Labs, Nano-LPS100) that is mounted on a *x-y* manual stage is installed on the microscope for cell locating and brightfield registration. The trans-illumination halogen lamp equipped with the microscope is used for collecting the brightfield images. The trans-illumination lamp power supply is connected to an analog/digital I/O board and controlled by a NI-DAQ card installed in the computer’s motherboard. This modification allows the computer to adjust the lamp intensity by sending an analog output voltage from 0 V to 5 V to the lamp power supply. A 637 nm laser (collimated from a laser diode, HL63133DG, Thorlabs) and a 405 nm laser (Crystal laser), are coupled into two single mode fibers and focused onto the back focal plane of the objective lens with 1.45 NA (UAPON 150XOTIRF, Olympus America Inc.) for data collection and photobleaching. A quad-band dichroic and emission filter set (LF405/488/561/635-A; Semrock, Rochester, NY) set was used for sample illumination and emission. Emission light was filtered using a band-pass filter (FF01-692/40-25, Semrock) and collected on an iXon 897 electron-multiplying charge-coupled device (EM CCD) camera (Andor Technologies, South Windsor, CT). The EMCCD gain was set to 100, and frames were 256 × 256 pixels (for each channel) with a pixel size of 0.1067 μm. Images were acquired at maximum camera speed (~17-ms exposure for 256 × 256 pixel region) and a total of 10,000–20,000 frames were collected. The emission path includes a relay optical system (two lenses with f = 100 mm), a band-pass filter (FF01-692/40-25, Semrock), and an EMCCD camera (iXon 897, Andor Technologies PLC.). All of the instruments are controlled by custom-written software in Matlab (MathWorks Inc.).

### Superresolution imaging

Cells were imaged in standard dSTORM imaging buffer [15] with enzymatic oxygen scavenging system and primary thiol: 50mM Tris, 10mM NaCl, 10% w/v glucose, 168.8 U/ml glucose oxidase (Sigma #G2133),1404 U/ml catalase (Sigma #C9332), and 20 mM 2-aminoethanethiol (MEA), pH 8.5. For samples prepared on 25 mm coverslips, the coverslip was mounted on an Attofluor cell chamber (A-7816, life technologies), in which 1.5 ml of the dSTORM imaging buffer is added, and the chamber was then sealed by covering with a clean 25 mm coverslip to prevent oxygen permeation into the buffer. The cell chamber was mounted on a custom designed chamber holder that can tightly fix the chamber in place, and the cell chamber was kept in the holder for the entire sequential imaging cycles. The chamber holder was then placed on the sample stage and pushed against one corner of the sample holder to ensure the chamber holder can be repeatedly placed at the same position without any rotation of the sample. For cells in 8-well chamber slides, the sample was covered with 0.4 ml of dSTORM buffer and no seal was used. The geometry of the 8-well chamber slide allowed for placement of the sample on the stage without any custom holders. After locating a target cell, the micrometer readings of the sample stage were recorded and regarded as the [*x*, *y*] coordinates of the cell and used for locating the same cell in the subsequent steps. A transmission cell image was taken and saved as a reference image, which was used for brightfield registration. During the data acquisition, a 637 nm laser at ~1.7 kW/cm^2^ is used. A set of 10,000 frames was taken at 60 Hz for the microtubule data and the data after fluorescence-reducing. For post-fluorescence-reducing data in Fig 2B and 2D, a 405 nm laser at 5 W/cm^2^ is used together with the 637 nm laser, and the 405 nm laser also is directed upward.

### Brightfield registration

The brightfield registration is based on the method described previously by McGorty et al. [34] and is used for both registering the target cell and the stage stabilization during image acquisition. The initial cell registering is done by recording a brightfield cell image as a reference image (S3A Fig). The subsequent re-registering of the same cell includes two steps: first, the reference image of the target cell is loaded into the controller GUI; second, the target cell is aligned to the reference image through an iterative aligning algorithm in *x*, *y*, *and z* positions. The stage stabilization is achieved by re-aligning the target cell to its reference image between every 2000 frames. The aligning algorithm is detailed as follows:

1. Find *z* shift and adjust: First, collect 21 brightfield images at *z* positions from -500 nm to 500 nm relative to the current position (set by eye) using 50 nm step size (S4B Fig); second, calculate the normalized cross correlation between each of those 21 individual images within the z-stack (S4C Fig) and the original reference image; third, fit the largest 9 cross correlation points to a second order polynomial (S4D Fig); fourth, move the stage to the *z* position to that corresponding to the estimated peak cross correlation value.
2. Find *x* and *y* shifts and adjust: first, take a brightfield image at the current stage position; find the *x* and *y* shifts between the current image and the reference image through the function *findshift* (DIPimage); third, move the stage to the position (*x*, *y*) that is adjusted by the shifts in *x* and *y*.
3. Repeat steps 1 and 2 until the adjustments in *x*, *y*, and *z* positions are less than set tolerances, which are 5 nm in *x*, *y* and 10 nm in *z*. Alignment is verified by generating an overlay image (S4E Fig).

### Photobleaching and NaBH_4_ quenching for fluorescence-reducing

The sample was removed from the microscope, but kept in the chamber holder. The chamber holder was placed on a rack to prevent the coverslip bottom from contacting any surface. This is helpful to avoid bubbles in the immersion oil, which could affect the brightfield registration. For photobleaching, the cells were washed four times with PBS and left in the PBS buffer. Then the sample was placed back on the microscope, and the chamber holder was pushed against the same corner of the sample holder. The target cell was located according to the recorded stage micrometer coordinates and then aligned with the reference image of the cell using the brightfield registration. The cell was illuminated by a 637 nm laser at ~1.7 kW/cm^2^ and a 405 nm laser at ~0.25 kW/cm^2^ simultaneously for 5 minutes. Both lasers were directed straight up out of the objective lens. After all imaged cells were photobleached, the sample was removed from the microscope and placed on a rack. The cells were then washed four times with PBS. For NaBH_4_ quenching, the cells were washed four times with PBS and then incubated with ~0.1% NaBH_4_ in PBS for 10 minutes. The cells were then washed four times with PBS.

### Superresolution image analysis, reconstruction, and overlay

The collected data is analyzed via a 2D localization algorithm based on maximum likelihood estimation (MLE) [40,59] using a Poisson noise model. To convert the pixel values into the photon counts, a CCD offset is subtracted from collected data and the result is multiplied by a gain factor [40]. The method described in Huang et al. [40] is applied to identify the single emitters in each frame. The method performs two filtering steps to reduce the Poisson noise and smooth out the data, then finds the pixel coordinates of local maxima and uses these as the centers of fitting regions. Each fitting region measured 9 × 9 pixels, corresponding to a size of 0.92 μm^2^ at the sample plane. All the fitting regions were fed into the 2D localization algorithm that maximize the likelihood function using a Newton-Raphson method to iteratively update the fitting parameters, which includes the *x* and *y* positions, the total photon counts, *I*, and the background photon counts, *bg*.

The localized emitters were filtered through thresholds of a maximum background photon counts at 50–100, a minimum photon counts per frame per emitter at 250, and a minimum P-value at 0.01 [40]. The accepted emitters are used to reconstruct the SR image. Each emitter is represented by a 2D-Gaussian with *σ*_*x*_ and *σ*_*y*_ equal to the localization precisions, which are calculated from the Cramér-Rao Lower Bound (CRLB) of each estimate, in *x* and *y* dimensions, and with unit intensity. The single-color SR images are color scaled in pixel value using the MATLAB colormap *hot*. The intensity of the un-labeled images after photodestruction in Figs 2 and 3B and S1–S3 Figs were scaled by a factor of 3 compared to the other SR images in order to make visible the small remaining signal.

The multi-color SR images were generated by assigning a 3-digit color index, [R, G, B], to each single-color SR image, and the color index is scaled for each pixel by a factor proportional to the pixel value while maintains each of its digits between 0 and 1. The color encoded SR images were then summed together, and the resulting color indices that are beyond [1, 1, 1] were normalized by the maximum digit value in each index. The 3-digit color indices used for generating the four-color overlay images in Fig 4 are [1, 1, 0] for clathrin, [0, 1, 0] for tubulin, [0.8, 0.2, 0] for actin, and [0, 0.5, 1] for EGFR, while for the two-color overlay images, the indices are [1, 0, 1] for magenta and [0, 1, 0] for green.

### Estimation of cross-talk

The cross-talk of sequential imaging using various methods of photodestruction was calculated by re-imaging after photodestruction. Briefly, HeLa cells were fixed and labeled with anti-α-tubulin-AF647 as described, and imaged using dSTORM. After the indicated method of photodestruction—either NaBH_4_ quenching or photobleaching with NaBH_4_ quenching—cells were placed back into imaging buffer and multiple cells were re-imaged using dSTORM. During re-imaging, we additionally included low intensity 405 nm illumination to determine any changes in cross-talk. Alignment and stabilization were performed to a reference image collected prior to acquisition of the initial image. The cross-talk is estimated to be the remaining percentage of localized emitters after photodestruciton (relative to the initial image) in ~30 subregions from six independent cells. Shown are histograms of the estimated cross-talk from these subregions for various methods of photodestruction (Fig 2, right).

### Estimation of overlay precision using α- and β-tubulin

The global shift between α- and β-tubulin structures was estimated using the ‘*findshift*’ function of the DipImage toolbox (http://www.diplib.org/) for a given region of interest (three independent cells are shown in Fig 3D, and S6 and S8 Figs). This estimates any global shift in the two structures that may result from error in overlay causing a unidirectional shift between the two labels. We further estimated local shifts between α- and β-tubulin structures that may be a result of overlay error and/or slight changes in microtubule structure between labeling/imaging steps. To estimate local shifts, two end points on an approximately linear fragment of microtubule from the SR image of β-tubulin were selected from top to bottom and with coordinates (*x*_1_, *y*_1_) and (*x*_2_, *y*_2_). Then all localizations from both α- and β-tubulin within the rectangle defined by the two points are selected and rotated with an angle equal to *tan*[(*y*_2_−*y*_1_)/(*x*_2_−*x*_1_)], so that the selected microtubule fragments are along the *x*-axis. Then the distributions of the localizations along the *y*-axis and within a *y*-range that includes the whole width of the microtubule are fitted to a Gaussian function (see Fig 3E and 3F and S4, S6, and S8 Figs). The shift between the two microtubule fragments from α- and β-tubulin is taken to be the shift between the mean of the two Gaussian fits (dash lines in Fig 3E and 3F and S5, S7, and S9 Figs). The microtubule fragments were selected throughout the region of interest in each cell (Fig 3D and S5, S7, and S9 Figs).

## Supporting Information

S1 Fig Photobleaching alone results in substantial cross-talk and is unsuitable for s-SR. HeLa cells were fixed and labeled with anti-α-tubulin antibody directly conjugated with AF647. Cells were imaged using dSTORM (left) and subsequently washed with PBS and subjected to photobleaching using high intensity 405 nm and 637 nm illumination (see Materials and Methods). After photobleaching, cells were re-imaged using dSTORM, and the reconstructed image shows non-negligible cross-talk (center). Subregion quantification shows cross-talk of ~7% and noticeable residual tubulin structure (right). Scale bars in original image (left), 2 μm; scale bars in small subregions (right), 1 μm. Note, the contrast in post-photodestruction images was scaled 3× to highlight the level of cross-talk. Cross-talk was estimated by calculating residual localization in small, ~2×2 μm, subregions after photo-destruction (see Subregion comparison, right column). Data for each histogram represents ~30 subregions taken from at least five independent cells.
(TIF)

S2 Fig Photolysis of a photocleavable fluorophore results in substantial cross-talk and is insufficient for s-SR imaging. HeLa cells were fixed and labeled with anti-α-tubulin antibody conjugated with photocleavable biotin followed by secondary labeling with NeutrAvidin-AF647. Cells were imaged as described and subsequently subjected to photolysis by 405 nm illumination. (A) Cells were subjected to high intensity 405 nm illumination, ~0.24 kW/cm^2^, for 20 minutes in dSTORM buffer to minimize the effect of photobleaching, followed by washing to eliminate cleaved fluorophores, and re-imaging. (B) Cells were subjected to low intensity 405 nm illumination, ~6 W/cm^2^, for 20 minutes in dSTORM buffer to minimize the effect of photobleaching, followed by washing to eliminate cleaved fluorophores, and re-imaging. Shown at right are zoomed regions (white boxes) illustrating the substantial cross-talk and noticeable tubulin structure after photolysis. Note, the contrast in post-photolysis images was scaled 3× to highlight the level of cross-talk.
(TIF)

S3 Fig Quantitative comparison of different photodestruction methods on sample labeled through photocleavable biotin (PC-biotin). HeLa cells were first labeled with anti-α-tubulin conjugated to PC-biotin, then labeled with NeutrAvidin-A647. (A) Photodestruction with NaBH4 quenching alone results in noticeable cross-talk from the original image. Quantification of residual localizations after photodestruction shows cross-talk of ~10%, with the histogram shown. (B) Photodestruction with both photobleaching/photolysis and NaBH4 quenching shows little detectable cross-talk. Subregion quantification shows cross-talk of ~0.1%, representing a ~100-fold improvement over NaBH4 alone. The 405 nm illumination during the photobleaching procedure (see methods in main text) will also cause photolysis through the PC-biotin, and hence, the photodestruction is significantly increased. However, the complexity of sample preparation with PC-biotin and the biotin related nonspecific bindings make it unfavorable in sequential imaging. Scale bars in original image (left), 2 μm; scale bars in small subregions (right), 1 μm. Note, the contrast in post-photolysis images was scaled 3× to highlight the level of cross-talk. Cross-talk was estimated by calculating residual localization in small, ~2×2 μm, subregions after photo-destruction (see Subregion comparison, right column). Data for each histogram represents ~30 subregions taken from at least five independent cells.
(TIF)

S4 Fig Brightfield alignment, registration and stabilization. (A) Before the first image is collected, a brightfield reference image is collected. (B) For alignment, 21 images are collecting spanning 1μm in the z-dimension, at equal spacing (50 nm increments). (C) Each of the 21 images is compared with the reference image using cross-correlation analysis. (D) Resulting cross-correlation analysis of each z-slice (blue circles) and Gaussian fit of the best nine points (red line). The sample is shifted in z to the maximum of the Gaussian fit, and the image is subsequently aligned in the *xy*-plane. (E) The resulting overlay of the reference image (red) and current image (green) for the optimal z position. This process is iterated until the set tolerance is reached (5 nm in *x-y*, 10 nm in *z*).
(TIF)

S5 Fig The localization distribution of microtubule segments in Fig 3. The localization distributions (open circles) from α-tubulin (blue) and β-tubulin (red) were fit to a Gaussian (solid line). The shift was calculated from the peak separation between the two Gaussian curves. Each subfigure is numbered for the corresponding box in Fig 3D.
(TIF)

S6 Fig Overlay of β-tubulin (red) and α-tubulin (green) in cell 2 (Fig 3). The local shifts (white arrow) of 18 microtubule segments (white boxes) are the shift along the direction perpendicular to the microtubule segment. They show a good agreement with the global shift (purple arrow). Scale bar 1 μm.
(TIF)

S7 Fig The localization distribution of microtubule segments in S5 Fig. The localization distributions (open circles) from α-tubulin (blue) and β-tubulin (red) were fit to a Gaussian (solid line). The shift was calculated from the peak separation between the two Gaussian curves. Each subfigure is numbered for the corresponding box in S5 Fig.
(TIF)

S8 Fig Overlay of β-tubulin (red) and α-tubulin (green) in cell 3 (Fig 3). The local shifts (white arrow) of 16 microtubule segments (white boxes) are the shift along the direction perpendicular to the microtubule segment. They show a good agreement with the global shift (purple arrow). Scale bar 1 μm.
(TIF)

S9 Fig The localization distribution of microtubule segments in S7 Fig. The localization distributions (open circles) from α-tubulin (blue) and β-tubulin (red) were fit to a Gaussian (solid line). The shift was calculated from the peak separation between the two Gaussian curves. Each subfigure is numbered for the corresponding box in S7 Fig.
(TIF)

S10 Fig Brightfield registration of four color sequential imaging. The brightfield cell images used for registration and alignment correspond to the cell in Fig 4. The four structures were sequentially imaged in the order of clathrin, α-tubulin, actin and EGFR. The peaks of the cross-correlation curves (top row) maintain around 0.95 with small decrease overtime. The bottom row shows the overlay images of the reference cell image (red) and the current cell image (green) during the brightfield registration for each structure.
(TIF)

S11 Fig Resting HeLa cells show no correlation between receptor distribution and clathrin structures. Following serum starvation, HeLa cells were fixed and labeled with an anti-EGFR-A647 antibody. dSTORM imaging was performed, and the reconstruction shows a near-uniform distribution of EGFR in the absence of activation. Notably, we observe no large aggregates of EGFR as observed after the addition of EGF (see Fig 5). (B) After photodestruction, cells were re-labeled with anti-clathrin-AF647 and imaged. (C-E) Insets highlighted in (A) and (B) show the distribution of EGFR (C), clathrin structures (D), and the overlay (E).
(TIF)
