# Supplementary figures and images for: Sequential Superresolution Imaging of Multiple Targets Using a Single Fluorophore
Source: PLoS One. 2015 Apr 10;10(4):e0123941. doi: 10.1371/journal.pone.0123941 (PMC4393115; doi:10.1371/journal.pone.0123941)

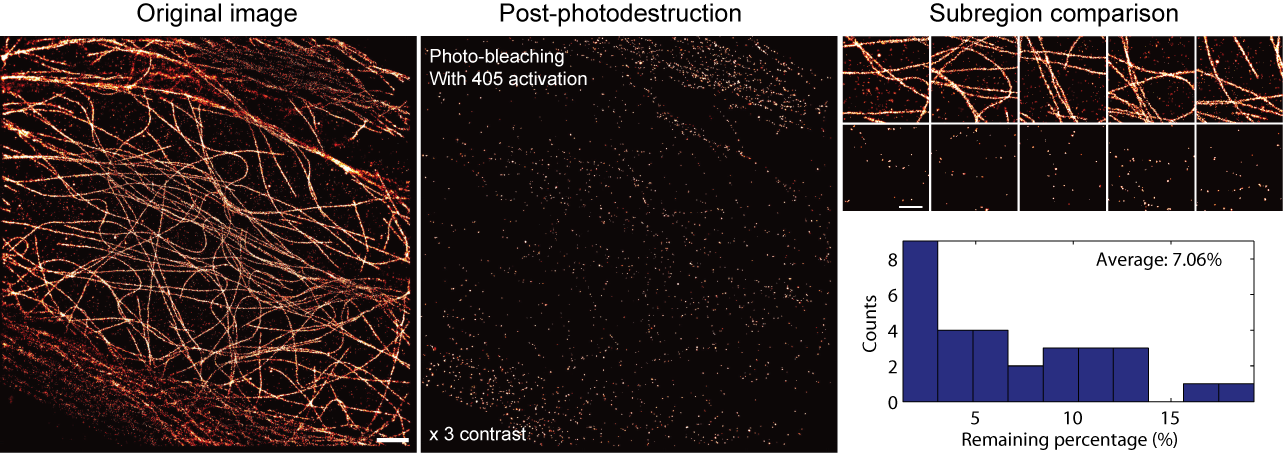

Supplement: S1 Fig — HeLa cells were fixed and labeled with anti-α-tubulin antibody directly conjugated with AF647. Cells were imaged using dSTORM (left) and subsequently washed with PBS and subjected to photobleaching using high intensity 405 nm and 637 nm illumination (see Materials and Methods). After photobleaching, cells were re-imaged using dSTORM, and the reconstructed image shows non-negligible cross-talk (center). Subregion quantification shows cross-talk of ~7% and noticeable residual tubulin structure (right). Scale bars in original image (left), 2 μm; scale bars in small subregions (right), 1 μm. Note, the contrast in post-photodestruction images was scaled 3× to highlight the level of cross-talk. Cross-talk was estimated by calculating residual localization in small, ~2×2 μm, subregions after photo-destruction (see Subregion comparison, right column). Data for each histogram represents ~30 subregions taken from at least five independent cells. (TIF) [file pone.0123941.s001.tif]

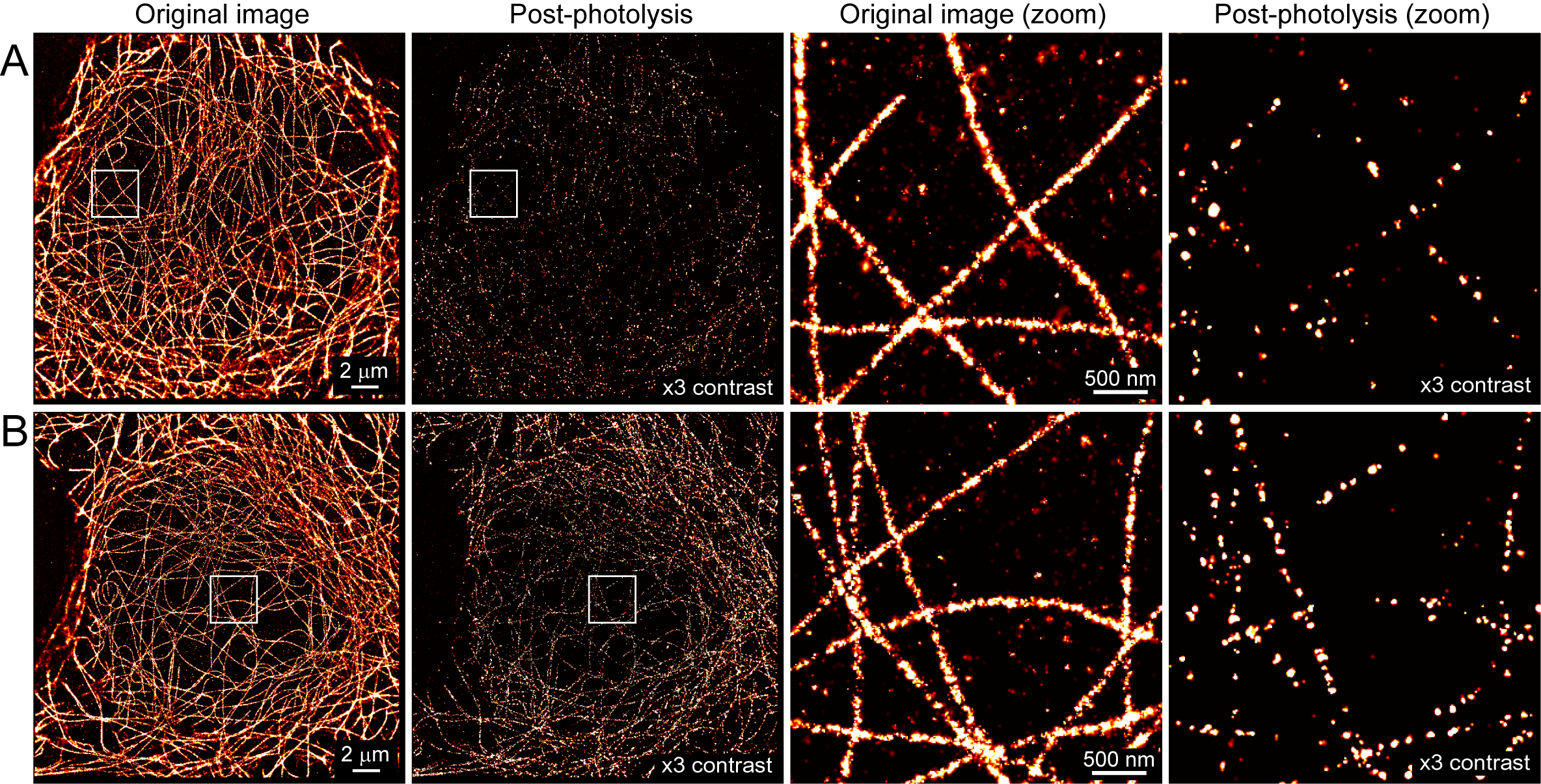

Supplement: S2 Fig — HeLa cells were fixed and labeled with anti-α-tubulin antibody conjugated with photocleavable biotin followed by secondary labeling with NeutrAvidin-AF647. Cells were imaged as described and subsequently subjected to photolysis by 405 nm illumination. (A) Cells were subjected to high intensity 405 nm illumination, ~0.24 kW/cm2, for 20 minutes in dSTORM buffer to minimize the effect of photobleaching, followed by washing to eliminate cleaved fluorophores, and re-imaging. (B) Cells were subjected to low intensity 405 nm illumination, ~6 W/cm2, for 20 minutes in dSTORM buffer to minimize the effect of photobleaching, followed by washing to eliminate cleaved fluorophores, and re-imaging. Shown at right are zoomed regions (white boxes) illustrating the substantial cross-talk and noticeable tubulin structure after photolysis. Note, the contrast in post-photolysis images was scaled 3× to highlight the level of cross-talk. (TIF) [file pone.0123941.s002.tif]

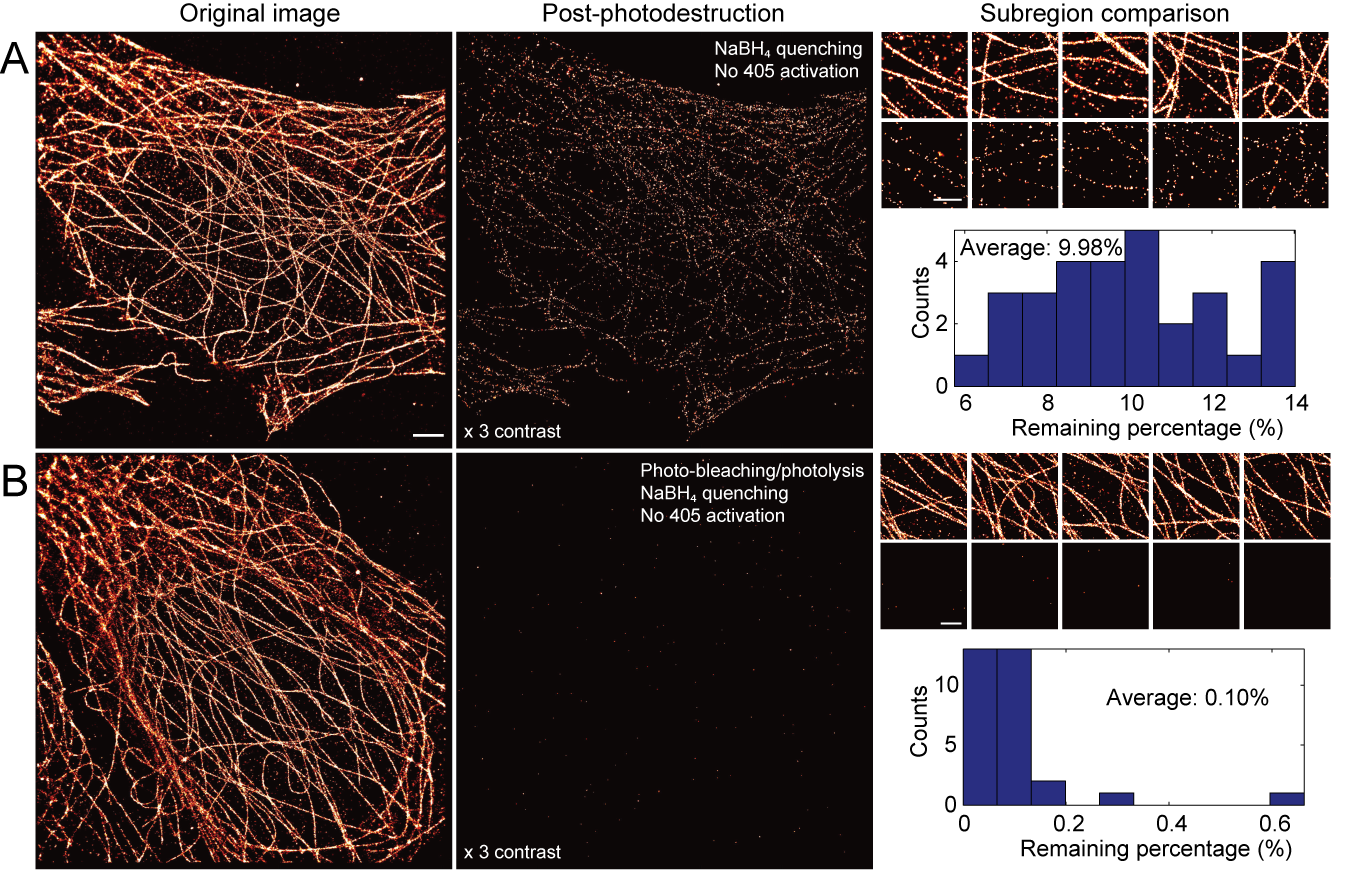

Supplement: S3 Fig — HeLa cells were first labeled with anti-α-tubulin conjugated to PC-biotin, then labeled with NeutrAvidin-A647. (A) Photodestruction with NaBH4 quenching alone results in noticeable cross-talk from the original image. Quantification of residual localizations after photodestruction shows cross-talk of ~10%, with the histogram shown. (B) Photodestruction with both photobleaching/photolysis and NaBH4 quenching shows little detectable cross-talk. Subregion quantification shows cross-talk of ~0.1%, representing a ~100-fold improvement over NaBH4 alone. The 405 nm illumination during the photobleaching procedure (see methods in main text) will also cause photolysis through the PC-biotin, and hence, the photodestruction is significantly increased. However, the complexity of sample preparation with PC-biotin and the biotin related nonspecific bindings make it unfavorable in sequential imaging. Scale bars in original image (left), 2 μm; scale bars in small subregions (right), 1 μm. Note, the contrast in post-photolysis images was scaled 3× to highlight the level of cross-talk. Cross-talk was estimated by calculating residual localization in small, ~2×2 μm, subregions after photo-destruction (see Subregion comparison, right column). Data for each histogram represents ~30 subregions taken from at least five independent cells. (TIF) [file pone.0123941.s003.tif]

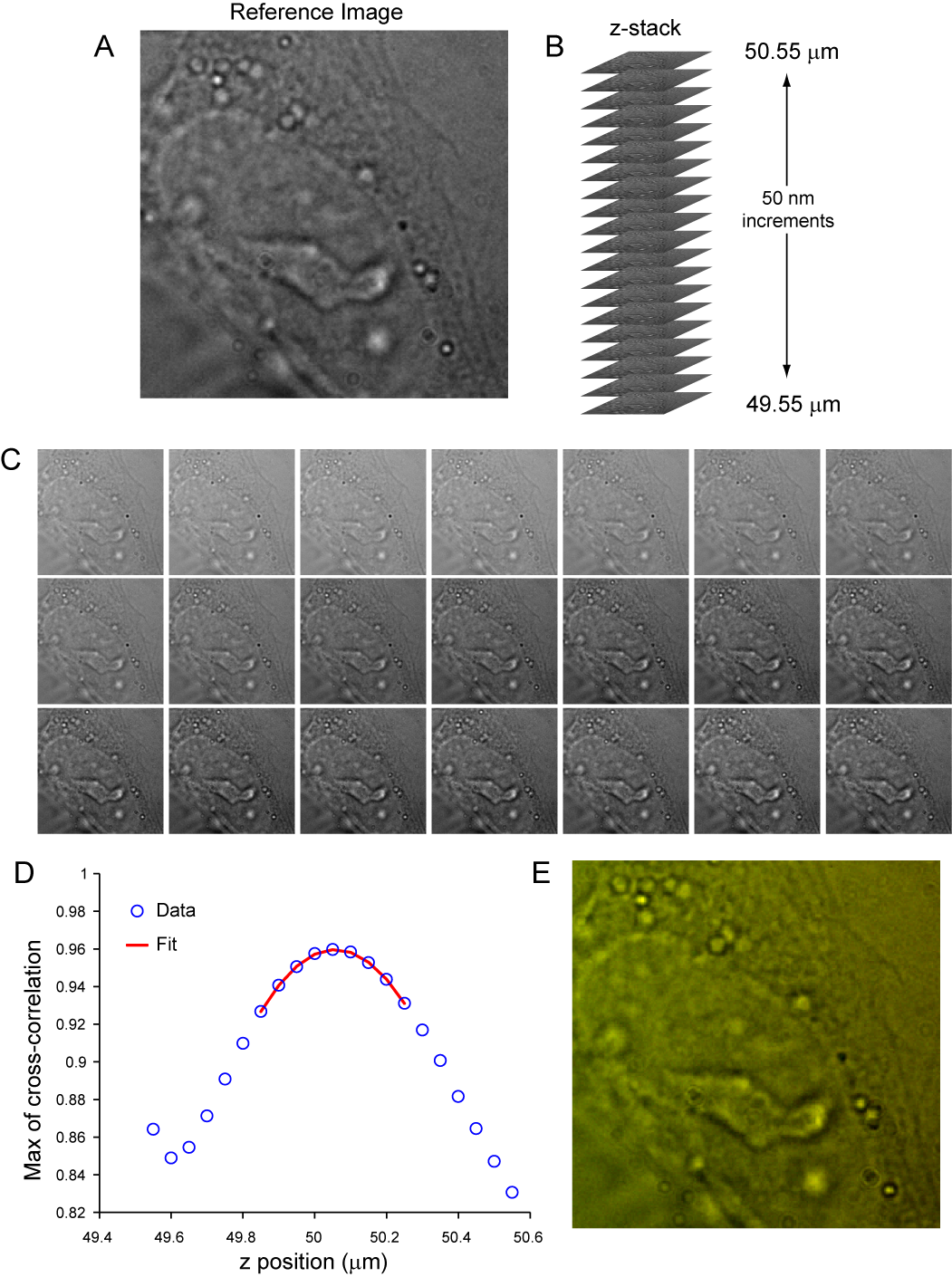

Supplement: S4 Fig — (A) Before the first image is collected, a brightfield reference image is collected. (B) For alignment, 21 images are collecting spanning 1μm in the z-dimension, at equal spacing (50 nm increments). (C) Each of the 21 images is compared with the reference image using cross-correlation analysis. (D) Resulting cross-correlation analysis of each z-slice (blue circles) and Gaussian fit of the best nine points (red line). The sample is shifted in z to the maximum of the Gaussian fit, and the image is subsequently aligned in the xy-plane. (E) The resulting overlay of the reference image (red) and current image (green) for the optimal z position. This process is iterated until the set tolerance is reached (5 nm in x-y, 10 nm in z). (TIF) [file pone.0123941.s004.tif]

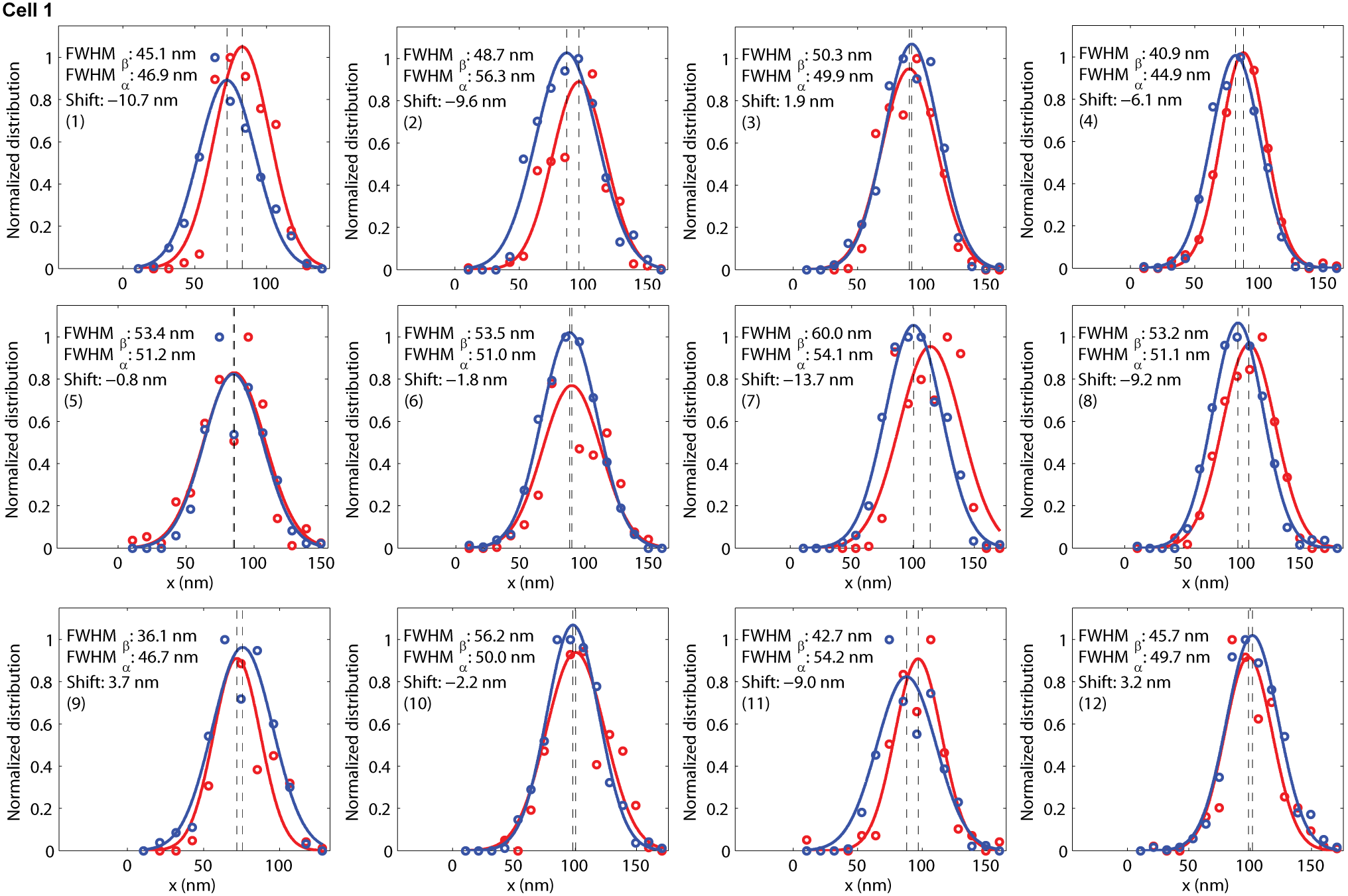

Supplement: S5 Fig — The localization distributions (open circles) from α-tubulin (blue) and β-tubulin (red) were fit to a Gaussian (solid line). The shift was calculated from the peak separation between the two Gaussian curves. Each subfigure is numbered for the corresponding box in Fig 3D. (TIF) [file pone.0123941.s005.tif]

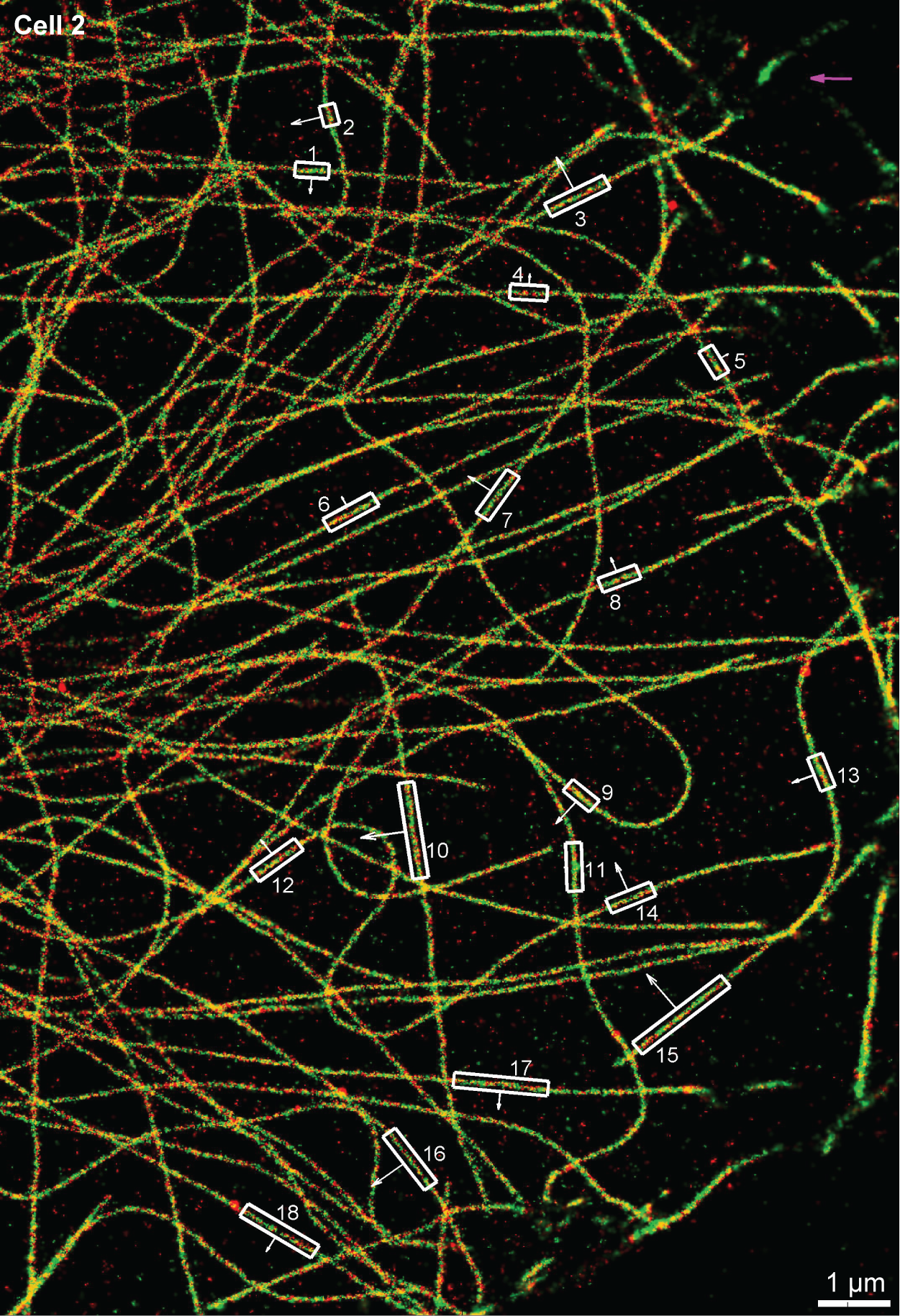

Supplement: S6 Fig — The local shifts (white arrow) of 18 microtubule segments (white boxes) are the shift along the direction perpendicular to the microtubule segment. They show a good agreement with the global shift (purple arrow). Scale bar 1 μm. (TIF) [file pone.0123941.s006.tif]

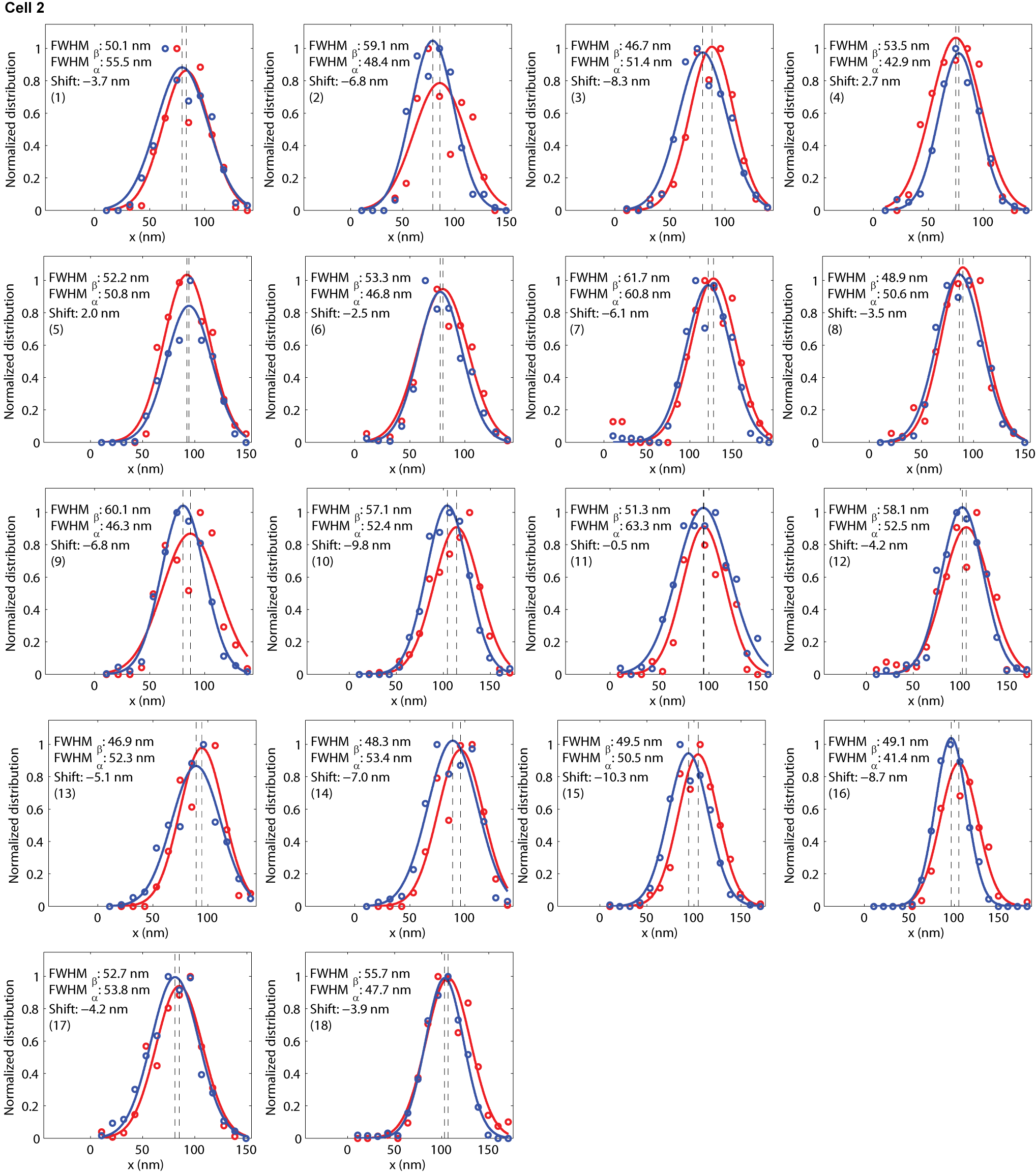

Supplement: S7 Fig — The localization distributions (open circles) from α-tubulin (blue) and β-tubulin (red) were fit to a Gaussian (solid line). The shift was calculated from the peak separation between the two Gaussian curves. Each subfigure is numbered for the corresponding box in S5 Fig. (TIF) [file pone.0123941.s007.tif]

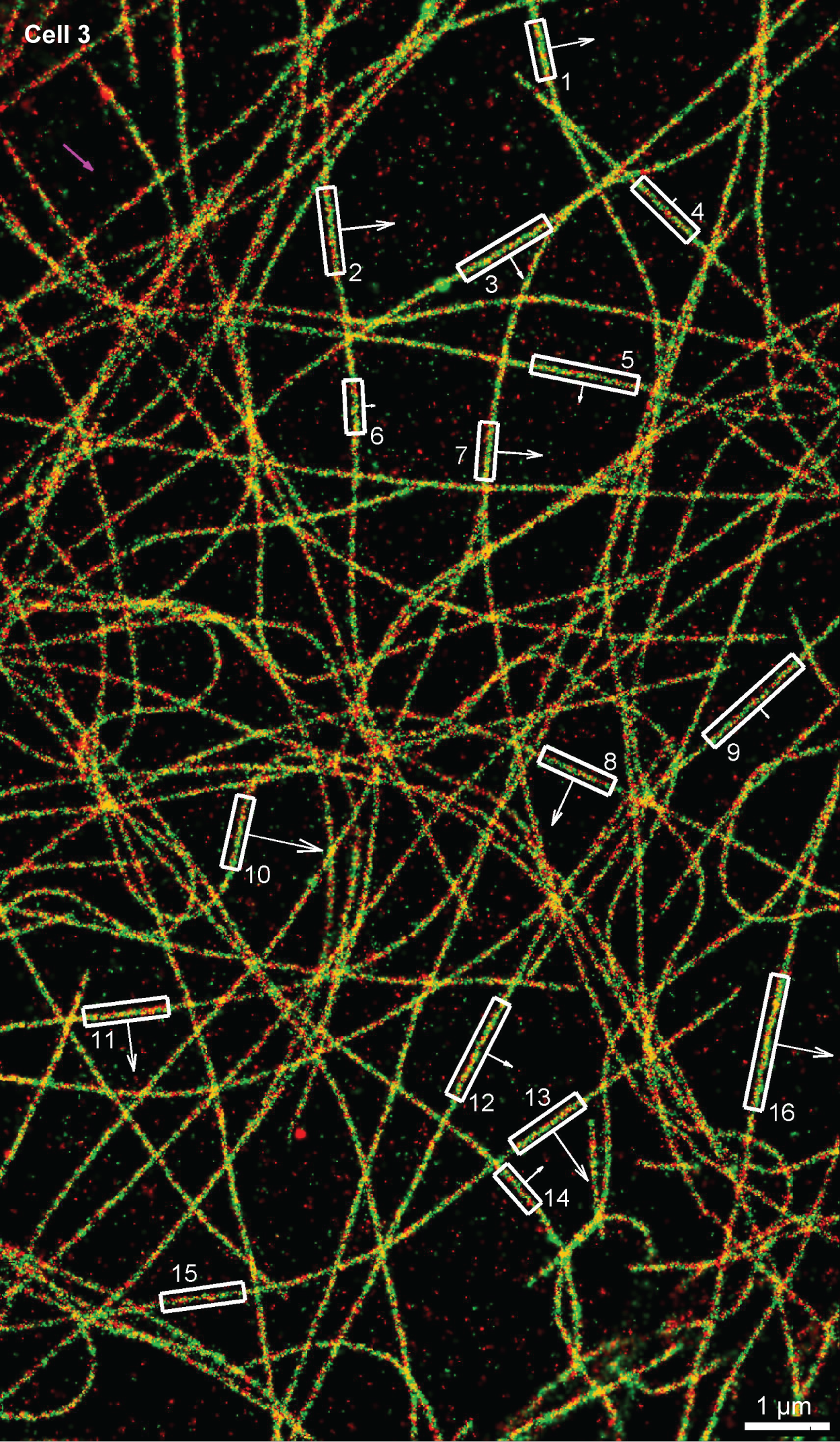

Supplement: S8 Fig — The local shifts (white arrow) of 16 microtubule segments (white boxes) are the shift along the direction perpendicular to the microtubule segment. They show a good agreement with the global shift (purple arrow). Scale bar 1 μm. (TIF) [file pone.0123941.s008.tif]

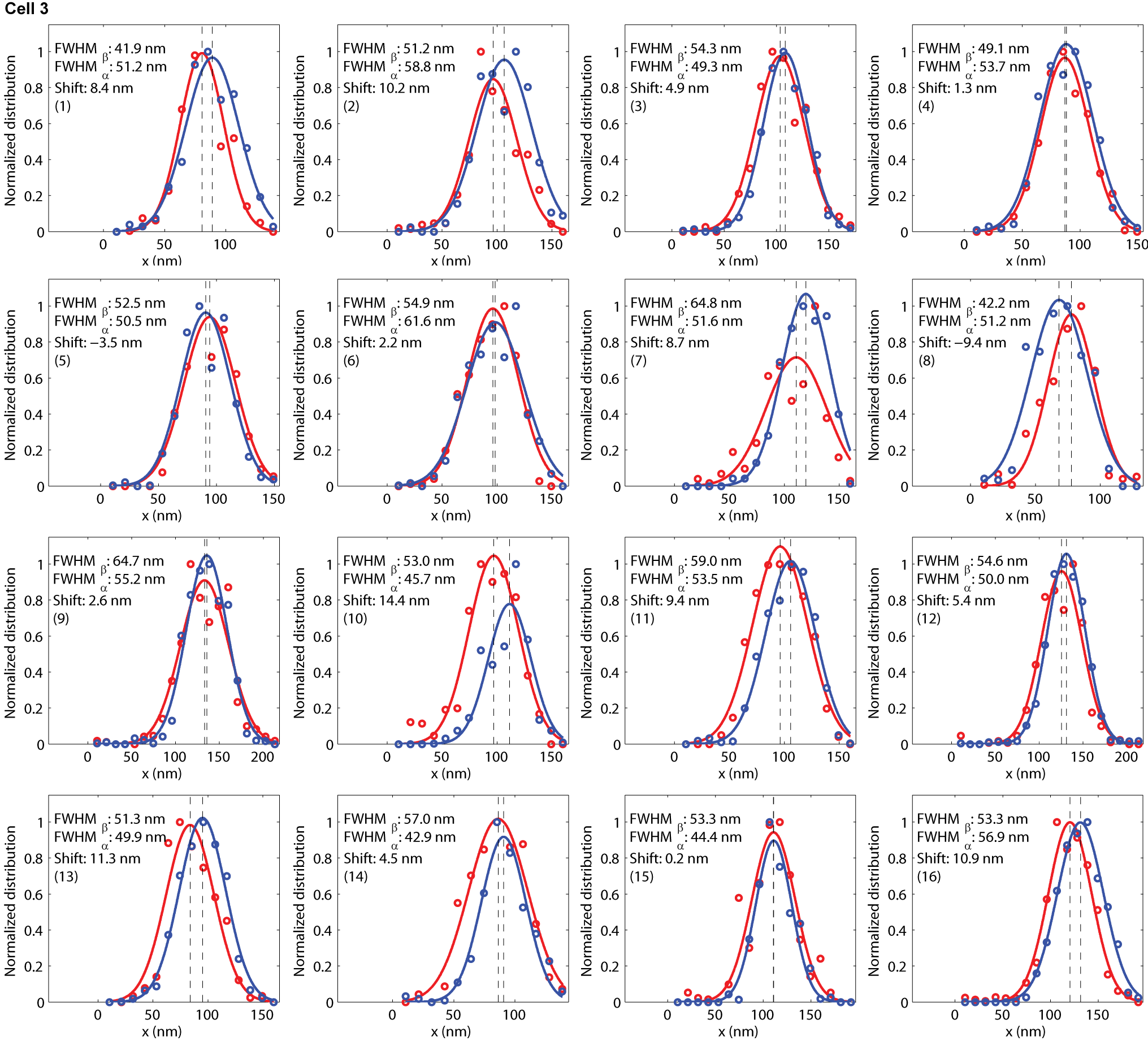

Supplement: S9 Fig — The localization distributions (open circles) from α-tubulin (blue) and β-tubulin (red) were fit to a Gaussian (solid line). The shift was calculated from the peak separation between the two Gaussian curves. Each subfigure is numbered for the corresponding box in S7 Fig. (TIF) [file pone.0123941.s009.tif]

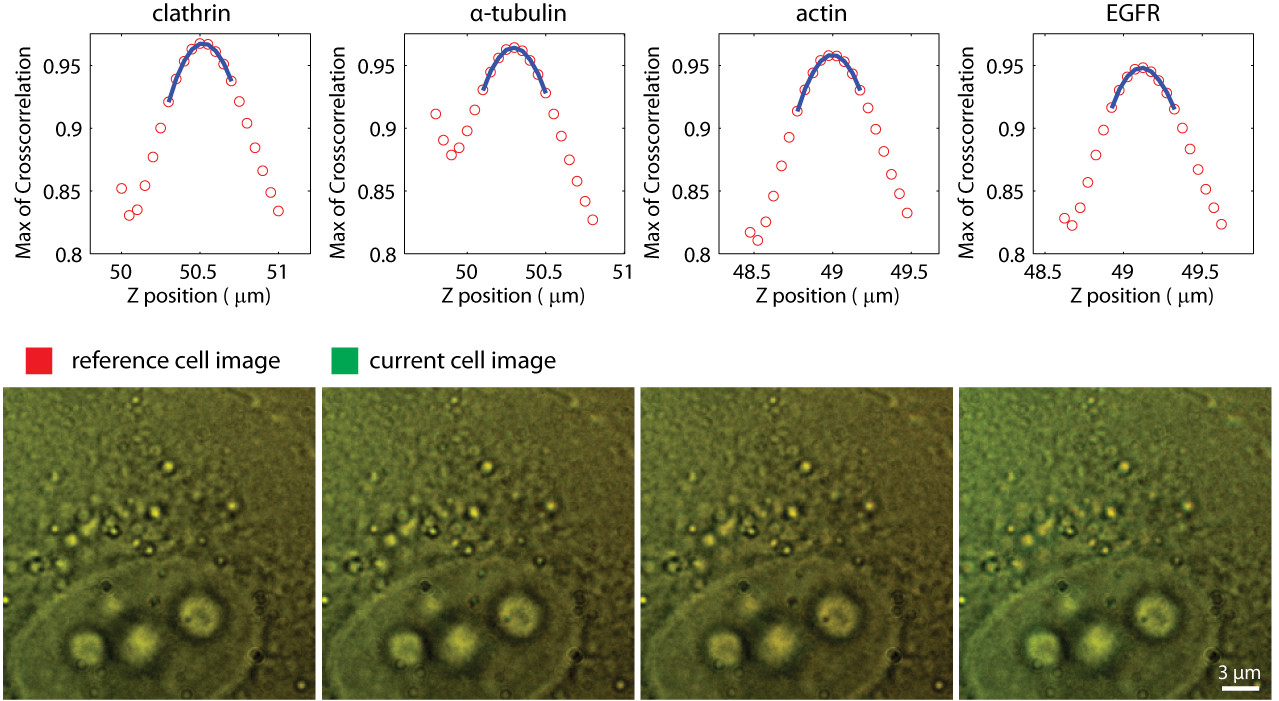

Supplement: S10 Fig — The brightfield cell images used for registration and alignment correspond to the cell in Fig 4. The four structures were sequentially imaged in the order of clathrin, α-tubulin, actin and EGFR. The peaks of the cross-correlation curves (top row) maintain around 0.95 with small decrease overtime. The bottom row shows the overlay images of the reference cell image (red) and the current cell image (green) during the brightfield registration for each structure. (TIF) [file pone.0123941.s010.tif]

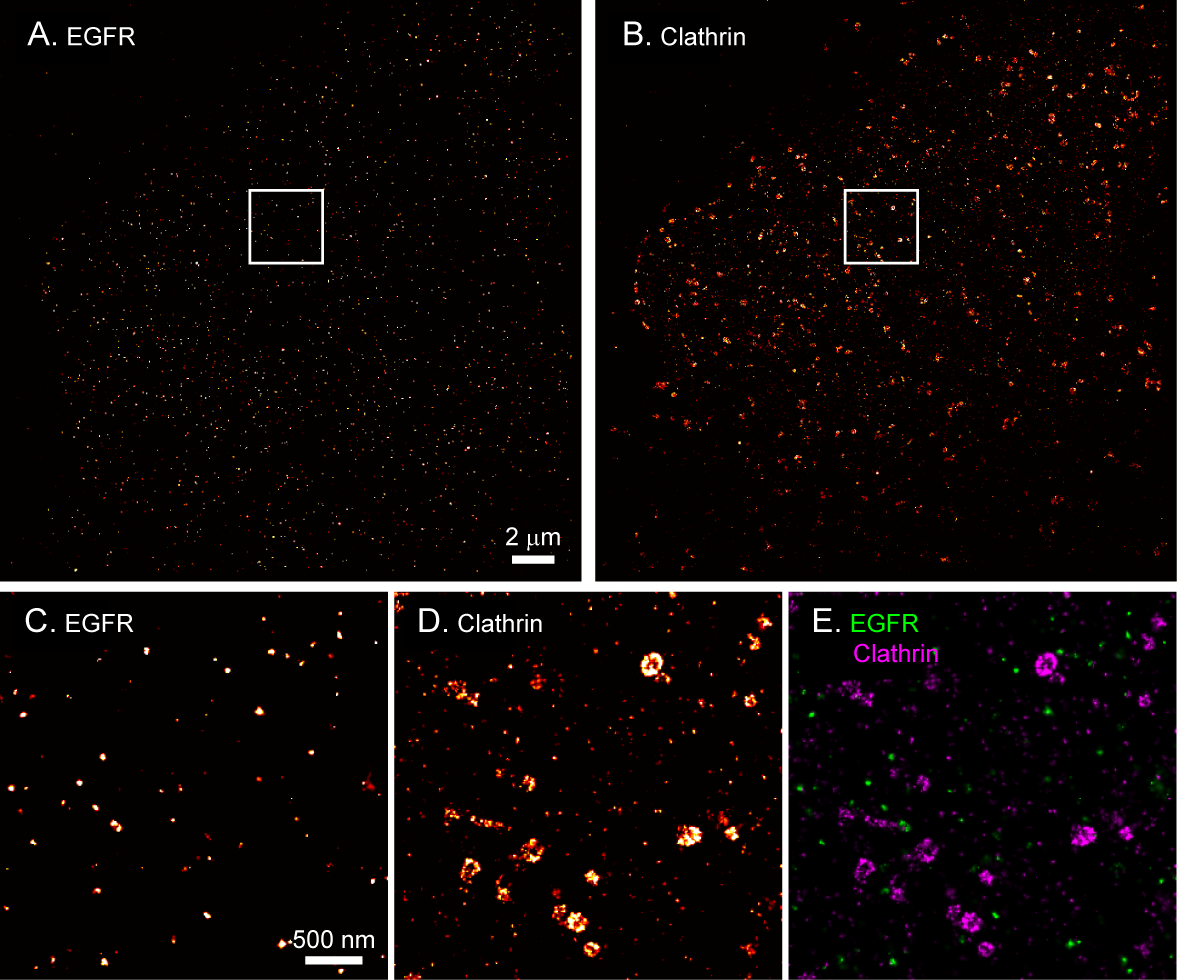

Supplement: S11 Fig — Following serum starvation, HeLa cells were fixed and labeled with an anti-EGFR-A647 antibody. dSTORM imaging was performed, and the reconstruction shows a near-uniform distribution of EGFR in the absence of activation. Notably, we observe no large aggregates of EGFR as observed after the addition of EGF (see Fig 5). (B) After photodestruction, cells were re-labeled with anti-clathrin-AF647 and imaged. (C-E) Insets highlighted in (A) and (B) show the distribution of EGFR (C), clathrin structures (D), and the overlay (E). (TIF) [file pone.0123941.s011.tif]
